# Extracting Diagnoses and Investigation Results from Unstructured Text in Electronic Health Records by Semi-Supervised Machine Learning

**DOI:** 10.1371/journal.pone.0030412

**Published:** 2012-01-19

**Authors:** Zhuoran Wang, Anoop D. Shah, A. Rosemary Tate, Spiros Denaxas, John Shawe-Taylor, Harry Hemingway

**Affiliations:** 1 Department of Computer Science, University College London, London, United Kingdom; 2 School of Mathematical and Computer Sciences, Heriot-Watt University, Edinburgh, United Kingdom; 3 Clinical Epidemiology Group, Department of Epidemiology and Public Health, University College London, London, United Kingdom; 4 Department of Informatics, University of Sussex, Brighton, United Kingdom; Dana-Farber Cancer Institute, United States of America

## Abstract

**Background:**

Electronic health records are invaluable for medical research, but much of the information is recorded as unstructured free text which is time-consuming to review manually.

**Aim:**

To develop an algorithm to identify relevant free texts automatically based on labelled examples.

**Methods:**

We developed a novel machine learning algorithm, the ‘Semi-supervised Set Covering Machine’ (S3CM), and tested its ability to detect the presence of coronary angiogram results and ovarian cancer diagnoses in free text in the General Practice Research Database. For training the algorithm, we used texts classified as positive and negative according to their associated Read diagnostic codes, rather than by manual annotation. We evaluated the precision (positive predictive value) and recall (sensitivity) of S3CM in classifying unlabelled texts against the gold standard of manual review. We compared the performance of S3CM with the Transductive Vector Support Machine (TVSM), the original fully-supervised Set Covering Machine (SCM) and our ‘Freetext Matching Algorithm’ natural language processor.

**Results:**

Only 60% of texts with Read codes for angiogram actually contained angiogram results. However, the S3CM algorithm achieved 87% recall with 64% precision on detecting coronary angiogram results, outperforming the fully-supervised SCM (recall 78%, precision 60%) and TSVM (recall 2%, precision 3%). For ovarian cancer diagnoses, S3CM had higher recall than the other algorithms tested (86%). The Freetext Matching Algorithm had better precision than S3CM (85% versus 74%) but lower recall (62%).

**Conclusions:**

Our novel S3CM machine learning algorithm effectively detected free texts in primary care records associated with angiogram results and ovarian cancer diagnoses, after training on pre-classified test sets. It should be easy to adapt to other disease areas as it does not rely on linguistic rules, but needs further testing in other electronic health record datasets.

## Introduction

Although electronic health records are an important source of data for health research, much of the information is stored in an unstructured way and can be difficult to extract. Research to date has predominantly used the coded data because it is readily analysed, but unstructured ‘free’ text in clinical entries may contain important information [1]–[4]. Manual review of free text is time-consuming and may require anonymisation to protect patient confidentiality. There has therefore been interest in software algorithms to analyse free text; examples include programs to identify angina diagnoses [2] and acute respiratory infections [4]. Analysis of clinical text is difficult because it can contain a wide range of terminology, complex language structures, context-specific abbreviations, and acronyms. Medical natural language processing systems such as MedLEE [5] rely on a detailed knowledge base and manually programmed linguistic rules. Natural language processors are expensive to develop as they have to be tuned specifically for each task or disease area.

Alternatively, a machine learning approach may be used, in which the computer attempts to ‘learn’ from a collection of training examples and apply this knowledge to classify new texts. For example, Support Vector Machine (SVM) algorithms have been used for a range of classification tasks based on electronic clinical notes, such as identifying smoking status [6], [7] and predicting response to quality of life questionnaires [8]. Hidden Markov Models have been used for paragraph-level topic segmentation and labelling in electronic health records [9], [10]. For the task of automatic diagnostic coding, cascade or hybrid systems with machine learning components have been shown to outperform purely rule-based or pattern matching systems [11]–[13]. The advantage of machine learning approaches is that they do not require manual programming of specific language features or knowledge of the subject area. However their performance can be variable, depending on the particular machine learning algorithm as well as the similarity between the underlying feature distributions in the training and the test sets.

Our aim was to develop a machine learning algorithm to classify whether a free text entry contains information of interest (e.g. a diagnosis or test result). Our novel algorithm, the ‘Semi-supervised Set Covering Machine’ (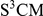) is related to two previous models by Rosales et al. [14]. Firstly they demonstrated a joint framework of semi-supervised active learning based on a Naïve Bayes Network and showed that unlabelled data in addition to the labelled training examples could contribute to the learning process. After this, in a separate work, they introduced an 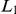-regularised SVM-style classifier, which enabled sparse feature representations for the target information to be obtained directly after learning [15].

We tested the 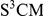 algorithm on free text samples from the UK General Practice Research Database (GPRD) which are relevant to our ongoing research studies. GPRD contains anonymised longitudinal medical records from 5 million patients actively registered in 590 contributing primary care centres [16]. It has been widely used for research on drug safety and clinical epidemiology [17]. It contains information on diagnoses, referrals, test results and prescriptions. Diagnoses are coded by general practitioners (GP) using the ‘Read’ coding system [18], and each Read coded entry may contain additional information as free text. This free text can contain clinical notes entered by the GP (e.g. test results, discussion with a patient, referral letters) as well as scanned clinic letters and discharge summaries.

We applied the 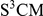 algorithm to an example of identifying texts containing investigation results (coronary angiograms) and an example of detecting diagnoses (ovarian cancer). Coronary angiograms are performed in hospital but are relevant to the long term management of patients with ischaemic heart disease in primary care. The longitudinal nature of the GPRD record is extremely useful for such studies but the coded record rarely contains angiogram results; only 4.2% of GPRD patients with myocardial infarction have a Read code stating the angiogram result, but a larger proportion have a code stating that an angiogram was performed. It is not possible to obtain angiogram results from hospital records for GPRD patients because they are anonymised to protect confidentiality. However, investigation results may be recorded in the free text in GPRD, either typed by the GP or in scanned letters. The Read codes associated with such texts may be non-specific (e.g. ‘Scanned letter’) so they are difficult to identify by conventional means.

The second case study aimed to detect suspected or definite diagnosis stated in the text prior to the date that it is formally coded. Ovarian cancer is a condition with insidious onset of symptoms, making it difficult to diagnose early, but documentation of suspected cancer may occur in the free text prior to a formal coded diagnosis [1]. This provides insight into the clinical reasoning of the doctor, and is relevant to research aimed at achieving earlier diagnosis in ovarian cancer.

We compared the performance of 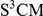 against three other algorithms: the original fully-supervised SCM [19], the Transductive Support Vector Machine (TSVM) [20] which is a semi-supervised but non-sparse algorithm, and the Freetext Matching Algorithm (FMA), a natural language processing system we have developed (see Text S1).

## Methods

### Ethics statement

The General Practice Research Database (GPRD) Division of the Medicines and Healthcare products Regulatory Agency has been granted Multi-Centre Research Ethics committee approval for all observational studies using GPRD data. All GPRD study proposals are prospectively reviewed by the GPRD Independent Scientific Advisory Committee, who specifically approved our study (protocols 07_069 and 09_123R) and did not require informed patient consent. All data including free text were anonymised by GPRD before being released to researchers.

### Development of machine learning algorithm

We developed a novel machine learning algorithm: the ‘Semi-supervised Set Covering Machine’ (‘
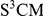
’). This utilised the feature of GPRD data that every free text entry is associated with a Read code. Clinical entries in the GP software are organised into ‘events’ which consist of a Read code denoting the diagnosis or context of the entry, and linked data fields for additional information or free text. GPs encode important diagnoses using Read codes so that they appear in a patient’s summary view and problem list. The text associated with Read codes for diagnoses may contain additional details about the diagnosis (e.g. qualifiers such as severity, or a narrative account), and is presented with the Read term on the doctor’s computer screen. Clinical information may also be entered in free text associated with non-specific Read codes such as ‘Scanned letter’ or ‘History/symptoms’; this can be more difficult to find, often requiring a search of the entire free text.

**Figure 1:**
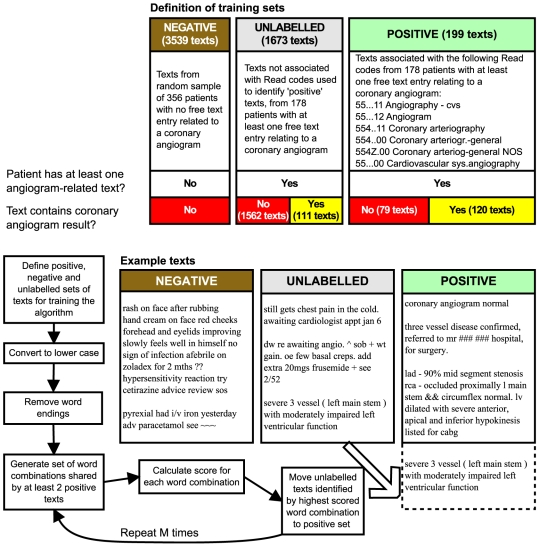
Semi-Supervised Set Covering Machine for detecting coronary angiogram results. Flow diagram showing logic of the 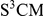 algorithm, and definitions of positive, negative and unlabelled training sets for detection of coronary angiogram results.

**Figure 2:**
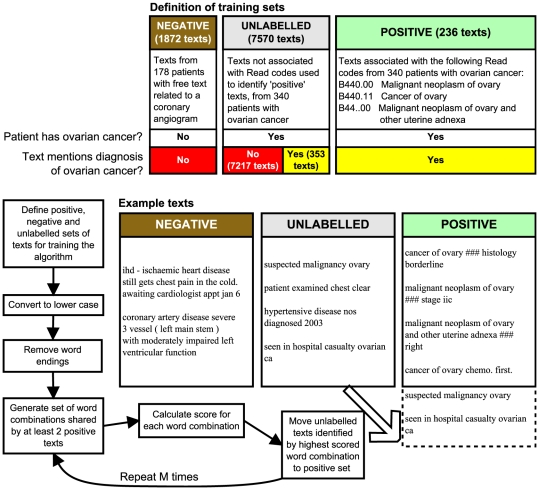
Semi-Supervised Set Covering Machine for detecting ovarian cancer diagnoses. Flow diagram showing logic of the 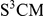 algorithm, and definitions of positive, negative and unlabelled training sets for detection of ovarian cancer diagnoses.

A set is defined mathematically as a collection of distinct objects in which the order of the objects does not matter. Two sets are considered to be identical if they contain the same objects. In this article we shall refer to sets of words as ‘word combinations’, free text entries associated with Read codes in GPRD as ‘texts’, and a set of texts used for training the algorithm as a ‘training set’. We defined ‘positive’, ‘negative’ and ‘unlabelled’ training sets as follows: the positive training set contained texts associated with the diagnosis of interest (identified by Read codes), the negative training set contained texts not associated with the diagnosis of interest, and the unlabelled set contained texts which the algorithm would try to classify. Figure 1 shows the definition of training sets for the coronary angiogram task, and Figure 2 for the ovarian cancer task. We compared the performance of the 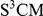 with other machine learning algorithms and with our Freetext Matching Algorithm (FMA). FMA uses tables of synonyms and hard-coded semantic information to map words and phrases in free text to Read terms, and assigns attributes for context (e.g. family history, negation or uncertainty). It is described in more detail in Text S1.

The 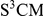 algorithm works by exploring combinations of words which are common to the texts of interest. Case, sentences, word endings and sentence structure are not considered. In the first stage, the algorithm compiles a list of all word combinations shared by at least two positive texts. Each word combination is scored on its ability to differentiate positive and negative texts (Figures 1 and 2). The algorithm then enriches its set of word combinations in an iterative manner using the positive and positively classified unlabelled texts. It is a ‘semi-supervised’ algorithm because it uses unlabelled as well as labelled texts during the training process. The unlabelled texts are used to hone the algorithm by enabling it to find additional word combinations which are associated with the diagnosis of interest, but which may not be included among the original ‘positive’ texts.

### Detailed technical description of Semi-supervised Set Covering Machine

**Notations and terminology.** We expected that only a small proportion of possible words found in the text would be of use in identifying texts of interest (i.e. the data are sparse) so we chose to use the set covering machine (SCM)[19] as our base algorithm, as it is suitable for sparse data. This algorithm was used in a semi-supervised manner, by training it on labelled and unlabelled texts in a bootstrapping technique.

We denoted each text a data point, 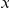, and assigned it a label 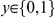. Texts with labels 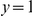 were called positive texts, and those with 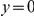 were called negative texts. In the case of semi-supervised learning, there was an unlabelled set with unknown labels 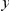, which would assist the algorithm during training and be labelled after the training. We used 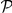, 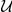 and 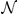 to represent the sets of positive, unlabelled and negative texts respectively. We defined a feature 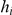 as a word or word combination (set of words). We expressed each text 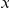 in terms of a feature vector as 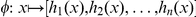, where the elements 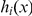 could have either binary or real values. Given a training set 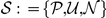, the goal of the algorithm was to find a predictive function 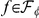 such that 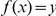. The pseudo-code for these algorithms is given in Figure S1.

#### Set Covering Machine (SCM)

The original SCM works in an iterative manner as follows. In each iteration, it greedily selects a feature 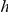 highest-scored by a score function, and removes the examples containing this feature before starting the next iteration, until all prospective (positive or negative) texts have been removed from the training set, or the size of the learned function 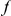 reaches a predefined value 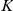. The feature components 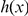 here are binary values, hence the predictive function 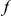 is in the form of a logical conjunction of a set of features. The score function is defined as the number of remaining positive (or negative) examples identified by the algorithm penalized by the number of unexpected examples identified. That is:
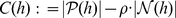
(1)where 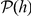 and 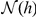 represent the respective subsets of the positive and negative examples that have feature 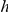, 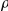 is a weight coefficient, and 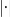 denotes the size of a set.

#### Modification of SCM for semi-supervised learning

To adapt the SCM to semi-supervised learning, we first added an additional penalty item to the score function, thus:
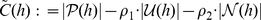
(2)where we used the 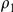-weighted number of the unlabelled examples that 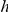 identifies (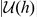) to give it an extra penalty, since there is a chance of identifying an unlabelled text that could be negative. The feature definition in our task was a combination of words, so the explicit feature vector of a text 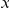 was the set of all possible word subsets that could be generated from the text.

To avoid dealing with exponentially large explicit vectors, our algorithm was designed as follows. First, it created a set of candidate features from the positive texts by extracting all word combinations shared by at least two texts, thus significantly reducing the feature space. We name this algorithm mSCM for the convenience of future discussion.

We used the algorithm in a semi-supervised manner, with a bootstrapping procedure to gain extra information from the unlabelled examples. In each bootstrap iteration, we moved the unlabelled texts identified by the mSCM in the previous iteration to the positive set, as ‘pseudo-positive’ texts. We trained a new mSCM based on the updated partitions of the dataset, and repeated this procedure 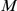 times, where 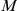 was a pre-defined number. In each iteration the mSCM compiled the common word combinations among the texts classified as positive, and appended them to the candidate feature set. Thus the algorithm could recall additional features that may not have been included during the initial run (which considered only the labelled positive texts).

The insight behind the bootstrapping procedure was that the unlabelled texts identified by the mSCM in each iteration had the possibility of being positive, and were therefore given a chance to contribute to the score function. Such positive contributions eliminate the penalty for the remaining unlabelled texts that share common features with them, and increase the possibility of selection of the features shared among them. However, as the pseudo-positive set grows, it increases the chance that the remaining unlabelled examples are identified as positive, and therefore increases the risk of false positives. Therefore we increased the penalty weight for unlabelled texts in each iteration by making it grow linearly with the size of the pseudo-positive set, as shown in Figure S1.

Compared to the work of Rosales et al. [14], [15], our 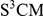 has the advantage of synchronously achieving sparse feature representation and contribution of unlabelled data, which were previously realised by two separate models. An advantage of our method compared to semi-supervised active learning is that it can use imperfectly labelled training examples based on diagnostic codes, thus avoiding the need to manually annotate the texts.

#### Implementation and complexity analysis

The algorithm was implemented in C++ and has been tested on Mac OS X and Linux (Ubuntu 11.04). Source code and documentation are available online (http://sourceforge.net/p/learnehr/home/Home/).

If we store each text record as a hashtable of words, the time complexity of checking whether a text record contains a word set 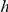 is 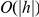. However, the most time-consuming step of the S
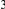
CM is the procedure for generating common word sets, which is performed in each bootstrapping iteration. Firstly, for two documents 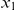 and 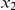 the time complexity of finding their largest common subset is 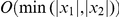. Let 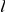 be the size of the largest common subset obtained. Then the enumeration of all common subsets will require 
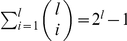
 unit operations. Although the time complexity is exponential, the running time would still be affordable in practice, as 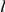 tends to be not too big. In practice, one could also restrict the maximum size of common word sets to a threshold 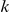, which would reduce the time complexity for enumerating all the features to 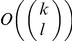.

### Testing

We tested the performance of the 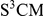 in identifying records containing angiogram test results and the diagnosis of ovarian cancer. The ‘gold standard’ was manual review by a medically qualified researcher who was blinded to the output of the algorithm. The angiogram texts were reviewed by a specialist registrar in internal medicine (AS) and the ovarian cancer texts by a gynaecological oncologist (AM).

We converted the free text examples to lower-case and removed the word endings using the FIHC stemmer [21] before applying the 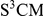. We defined precision (positive predictive value) as the proportion of texts labelled as positive which were true positives, and recall (sensitivity) as the proportion of all positive texts which were correctly labelled as positive by the algorithm.

We compared the performance of 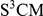 with a non-sparse semi-supervised algorithm called the Transductive Support Vector Machine (TSVM) [20], the original fully supervised SCM, and, for the ovarian cancer dataset, our FMA natural language processing system. We did not use FMA on the angiogram dataset because it only detects diagnoses which correspond to Read terms, and there are very few Read terms describing angiogram results. We tuned the parameter settings of the models (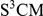, TSVM and SCM) based on the leave-one-out cross-validation (LOO-CV) method. Our adaptation of the LOO-CV method for semi-supervised learning was as follows. We removed the label of one positive text in turn to make it an unlabelled text, trained the algorithm based on this modified data set and tested its classification result for the pre-selected text. This process was applied to every positive text to obtain an average LOO-CV error rate.

We also evaluated the precision and recall of 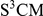 on classification at the patient level, compared to using Read codes only. We randomly split the case and control patients into a training set and a test set (50 cases and 100 controls for angiogram data; 100 cases and 50 controls for ovarian cancer) and repeated the experiment 10 times. We investigated the timing of the earliest angiogram result or diagnosis of ovarian cancer as detected by the algorithm or Read codes.

**Coronary angiogram dataset**

The GPRD Group maintain a library of free text records which have been pooled from previous anonymisation studies. Cases were identified as patients having at least one pre-anonymised freetext record in the library related to a coronary angiogram. Controls were randomly selected from the remaining patients who had at least one entry in the library of pre-anonymised freetext records. Two controls were matched to each case by age within 5 years. The test dataset comprised all pre-anonymised free text entries for the selected patients.

The case data consisted of 2090 free text entries from 178 patients from 122 practices. After removal of blanks and duplicates, 1872 texts remained, of which 199 had a Read code for a coronary angiogram (code list in Figure 1). We reviewed these texts manually and identified 231 records which contained angiogram results, of which 120 were associated with a Read code for angiogram (Table 1). The control data consisted of 3539 records, none of which had a Read code for a coronary angiogram.

**Table 1:** Selection of free text entries for training the algorithm.

	Coronary angiogram dataset	Ovarian cancer dataset
Number of patients	178 patients with at least one text relating to a coronary angiogram	340 patients with new diagnosis of ovarian cancer
Initial number of texts	2090	7860
Number of texts after removal of blanks and duplicates	1872	7806
Text together with Read term for analysis	No	Yes
Number of texts with positive Read code (positive training set)	199 texts with Read code for angiogram	236 texts with Read code for ovarian cancer
Number of texts with positive Read code and positive text on manual review	120 with angiogram results in text and Read code for angiogram	236 (all ovarian cancer Read terms regarded as positive)
Number of unlabelled texts which are positive on manual review	111	353
Number of unlabelled texts which are negative on manual review	1562	7217
Total number of unlabelled texts	1673	7570

Texts associated with Read codes for angiogram (n = 199) were taken as positive for the purpose of training the algorithm, whether or not they actually contained angiogram results. Texts from control patients were used as negative examples, and the remaining texts (n = 1673) from case patients were taken as unlabelled examples.

We compared the 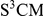 algorithm (with parameter settings: 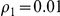, 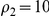, 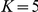 and 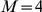) with TSVM (with regularization coefficient 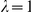, unlabelled data influence parameter 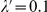 and positive class fraction of unlabelled data r = 0.1), and the fully supervised SCM (with 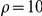).

#### Ovarian cancer dataset

The ovarian cancer dataset was from a study by Tate et al. investigating the dating of diagnosis of ovarian cancer in the GPRD [1]. The case selection criteria have been described previously [22] and are briefly reported here. The target population consisted of women between the ages of 40 and 80 from a random sample of 127 GP practices contributing to GPRD. From this population, we identified women aged 40 to 80 years, who were registered with the practice on 1 June 2002, and who had an incident diagnosis of ovarian cancer between 1 June 2002 and 31 May 2007 (recorded using a Read code in Figure 2). We excluded patients who were registered with the practice for less than 2 years or had a previously recorded Read code for ovarian cancer. We obtained anonymised free text records for all consultations recorded during the 12 months before the date of the earliest Read code indicating a referral for, or suspicion of, ovarian cancer, up to and including the date of definite diagnosis. The initial search yielded 7860 clinical events, from which we excluded blanks and duplicates.

Our test set consisted of 7806 clinical events with non-blank free text entries. The final number of patients was 340 (4 patients met the criteria for inclusion but had no free text recorded). Although all patients had a Read code for ovarian cancer in their electronic patient record, only 236 Read codes (from 234 patients) were associated with non-blank free text and were included in our sample. We manually reviewed texts containing the fragments ‘ov’, ‘ovar’ or ‘ov.’, and assigned them as ‘positive’ if they stated a suspected or definite diagnosis of ovarian cancer for the current patient. All other texts were assigned as ‘negative’, including those which mentioned ovarian cancer in another context (e.g. negation, family history or patient anxiety). We found 353 texts which referred to ovarian cancer but did not have a Read code for ovarian cancer (Table 1).

We trained the 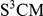 algorithm (with parameters 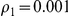, 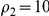, 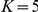 and 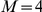) with the following training datasets: texts with Read codes for ovarian cancer (n = 236) were positive examples, texts without Read codes for ovarian cancer (n = 7570) were the unlabelled examples, and texts from angiogram case data (n = 1872) were negative examples (as we did not have access to control data for this study). For this test we appended the free text to the Read term of each record to make it more informative, and appear similar to the way it would be displayed on the GP computer system. We also tested the supervised SCM (with 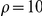), TSVM (with 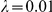, 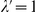 and 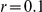) and the Freetext Matching Algorithm. FMA mapped the texts onto Read codes with a context attribute; for this test a Read code in Figure 2 was considered positive as long as it was not associated with an attribute for negation or family history.

## Results

### Coronary angiogram results

**Figure 3:**
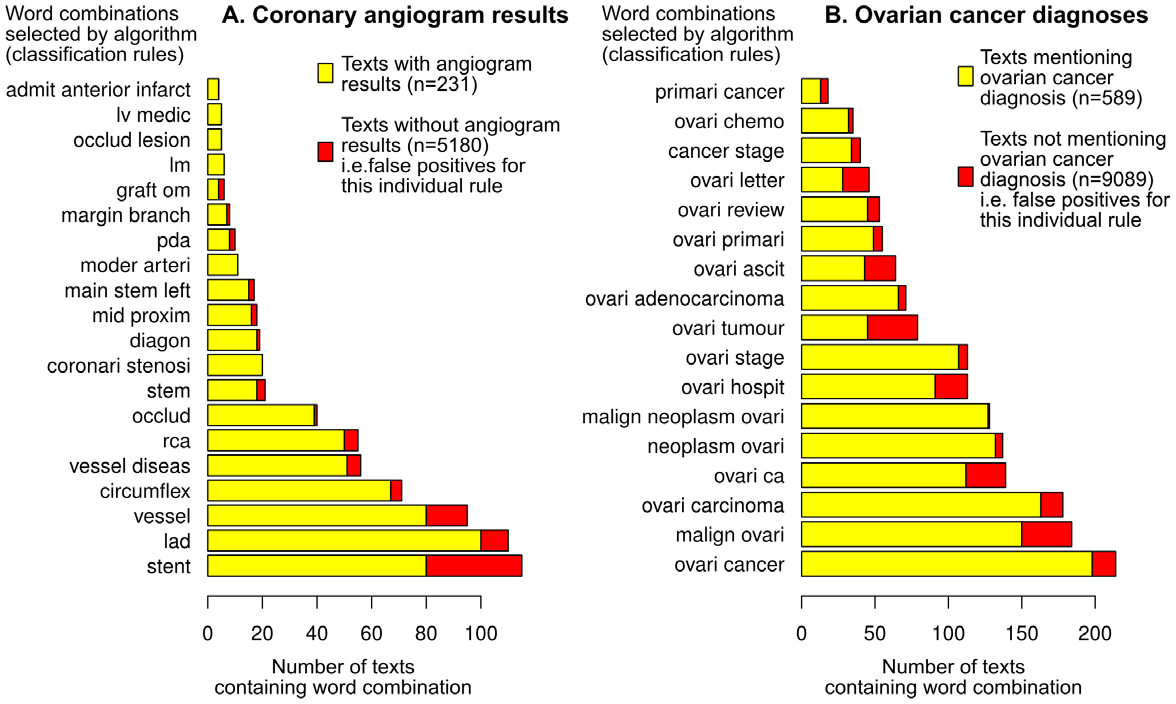
Word stem combinations extracted from free text records. List of word stem combinations selected as classification rules by 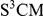 for (A) coronary angiogram and (B) ovarian cancer test sets. The bars show the frequency of each rule among the combined positive, negative and unlabelled training sets. Words were stemmed in order to aid the grouping of similar words; for example ‘ovarian’, ‘ovary’ and ‘ovaries’ were all converted to the common stem ‘ovari’.

Only 60% of texts in the ‘positive’ training set (with read codes for angiogram) actually contained angiogram results in the free text; some contained uninformative text such as ‘hospital admission’. However when tested on unlabelled texts, the 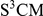 algorithm achieved 87% recall with 64% precision. It performed better than the TSVM (precision 3%, recall 2%) and the fully-supervised SCM (precision 60%, recall 78%; see Table 2). The most common word stems associated with positive texts were ‘vessel’, ‘stent’ and ‘lad’ (abbreviation for left anterior descending coronary artery; see Figure 3 A).

**Table 2:** Results of testing: classification of unlabelled texts.

Algorithm	Number of texts	True positive	False positive	False negative	Precision, % (95% CI)	Recall, % (95% CI)	F score, %
Presence of coronary angiogram results							
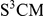	1673	96	55	15	63.6 (55.3, 71.1)	86.5 (78.4, 92.0)	73.3
SCM	1673	67	19	44	77.9 (67.4, 85.9)	60.4 (50.6, 69.4)	68.0
TSVM	1673	2	64	109	3.0 (0.5, 11.5)	1.8 (0.3, 7.0)	2.3
Ovarian cancer diagnosis							
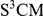	7570	303	106	50	74.1 (69.5, 78.2)	85.8 (81.7, 89.2)	79.5
FMA	7570	218	38	134	85.2 (80.1, 89.2)	61.9 (56.6, 67.0)	71.8
SCM	7570	95	53	254	64.2 (55.9, 71.8)	27.2 (22.7, 32.3)	38.2
TSVM	7570	26	534	323	4.6 (3.1, 6.8)	7.4 (5.0, 10.9)	5.7

Precision (positive predictive value) is the percentage of texts positively classified by the algorithm that are true positive, and recall (sensitivity) is the percentage of all positive texts correctly classified as positive by the algorithm. F score is the harmonic mean of precision and recall. Figures in parentheses are 95% confidence intervals.

In the patient level classification test, we found that the 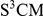 had higher precision than Read codes in identifying patients who had angiogram results (89% versus 71%), but recall was over 90% with both methods. The 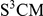 incorrectly detected angiogram results in 2.7% of control patients (Table 3).

**Table 3:** Results of testing: detection rate by patient of presence of angiogram results or ovarian cancer diagnosis in the free text.

Method	Precision (%)	Recall (%)	F score	Control error rate (%)
Presence of coronary angiogram results				
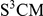	89.3 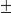 10.6	93.1 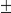 7.5	91.1 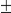 7.5	2.7 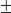 3.8
Read code	70.5 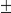 9.8	95.9 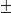 6.3	81.1 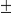 6.2	0
Ovarian cancer diagnosis				
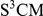	96.4 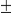 6.2	98.8 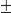 2.6	97.5 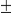 3.9	0
Read code	100	82.4 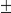 9.7	90.3 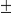 5.7	0

Mean 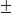 two standard deviations from 10 experiments testing 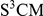 on classification at the patient level by splitting patients randomly into a training set and a test set.

Four patients had angiogram results in the free text earlier than the first angiogram Read code, and 43 patients had angiogram results in the free text but no Read code for angiogram anywhere in their record. Forty of these 47 patients were correctly identified by the algorithm. However, 15 records were incorrectly identified as containing angiogram results, giving precision 73%, recall 85% and F score 79%.

### Ovarian cancer diagnosis

The 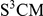 algorithm performed better than the other machine learning approaches in identifying diagnoses of ovarian cancer in unlabelled texts, detecting 303 of the 353 diagnoses (recall 86%, precision 74%). FMA had greater precision than the 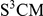 (85%) but lower recall (62%; see Table 2). The most common word stem combinations denoting a diagnosis of ovarian cancer were ‘ovari’ with either ‘cancer’, ‘malign’ or ‘carcinoma’ (Figure 3 B).

The algorithm identified 99% of the patients in the test set as having ovarian cancer, even though only 82% of patients had a Read code for ovarian cancer amongst the clinical entries in our dataset (Table 3).

Of the 138 free text records containing a diagnosis of ovarian cancer earlier than the first Read code for ovarian cancer, 123 were correctly identified by the algorithm. However, 81 records were incorrectly identified as denoting an ovarian cancer diagnosis, giving precision 60%, recall 89% and F score 72%.

### Performance

In the unlabelled text classification experiment, running on a Mac computer with an Intel Core i7 2.7 GHz processor and 4GB memory, the 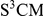 took on average 34.3s and 93.6s CPU time in each bootstrapping iteration on the angiogram data and the ovarian cancer data, respectively. Four bootstrapping iterations in total were performed each time to obtain the results in Table 2.

## Discussion

### Summary of main findings

We have developed a novel sparse semi-supervised learning algorithm to classify clinical text records, and have obtained promising results in pilot studies for identification of angiogram results and diagnoses of ovarian cancer in samples of free text from the GPRD. The algorithm performed well despite the fact that for the angiogram dataset, the allocation of training examples was imperfect. ‘Positive’ training examples were denoted by Read codes and not by manual review, and almost 40% of the texts with Read codes for angiogram did not actually contain angiogram results.

A strength of our algorithm is that the training examples can be provided by a diagnostic code search rather than requiring manual review. The algorithm does not rely on a pre-programmed knowledge base or linguistic rule set, and is easy to adapt to other subject areas or languages. It explores the unlabelled data as well as using the positive and negative sets, and compiles a comprehensive list of word combinations pertaining to the condition of interest, which may be used to feed further research.

The trade-off between recall and precision depends on the task; for example if the algorithm is used to select texts for anonymisation and manual review, good recall is more important than precision. Our Freetext Matching Algorithm achieved better precision than 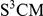 in detecting ovarian cancer diagnoses, but at the cost of only 62% recall. This is because FMA looked for phrases representing diagnoses which could be converted to Read terms, and might miss a diagnosis if the words ‘ovary’ and ‘cancer’ were widely separated. However, such texts might be recognised by 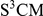, which ignores word order.

### Limitations of the 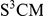 algorithm

The main limitation of our algorithm is that it does not use any language knowledge to aid interpretation of texts. As with other machine learning approaches, errors may occur through failure to recall texts containing rare or complex language expressions. Our algorithm attempts classification based only on commonly occurring sets of words, and its precision may be limited by incorrect inclusion of negated phrases. Punctuation, case and the order of words are also ignored; thus it does not utilise all the information that would be available to a human reviewer. Mis-spellings and abbreviations can also cause errors.

### Limitation of development and testing methodology

Although our testing methodology had strengths – use of two different tasks (detection of diagnosis and detection of a test result) on two different diseases (coronary artery disease and ovarian cancer) – the calculated precision and recall must be used with caution when extrapolating to other datasets. The performance of the algorithm will depend on the disease, the selection procedure for the training datasets, and the size of these datasets. Another limitation is that we only tested the algorithm on data from the GPRD. We recommend that if this algorithm is used for future studies, a sample of the results for each study should be reviewed manually to quantify precision and recall.

A general limitation of using free text is that patients with similar medical histories may have different amounts of information in the free text, influenced by the doctor's documentation habits and whether the GP practice routinely scans all correspondence. Researchers should assess the completeness of recording for a particular study and consider limiting the analysis to practices with more complete recording, or use statistical methods to account for missing data. However this limitation may diminish in the future as information technology becomes more widely adopted.

### Clinical and research application

Our approach may facilitate research using electronic health records where diagnoses or other information of interest (e.g. angiogram results) are recorded in free text rather than in coded form. The algorithm is semi-automatic and therefore cheap to run, and is fairly sensitive at identifying relevant texts. Although it is not accurate enough for definitive classification, it may be useful for filtering large databases to extract a smaller subset of texts for further analysis.

Although our test sets were from GPRD, this approach can be used on other sources of electronic health information such as discharge letters and electronic hospital notes. 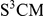 is not disease-specific and requires only a small amount of labelled data for training, because it gains additional information from unlabelled data. The only aspect of the algorithm that is language-specific is the ‘stemmer’ program which standardises word endings prior to analysis. 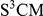 processes sets of words without regard to language features such as grammar or word order, so in principle it should work with many languages, including other Indo-European languages. However, for languages in which long compound words convey a complex meaning it may be necessary to split the words into individual morphemes (the smallest part of a language which has meaning on its own) and allow soft matching of those morphological variants when generating rules in 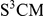 [23].

Future work will involve tuning the algorithm to be able to return more detailed information rather than the merely the absence or presence of a condition. We are working on a system to extract the number of diseased vessels from angiogram reports. We also aim to optimise the code and run it on larger datasets.

Future clinical uses of this algorithm in electronic health record systems may include assisting the coding process and auditing the quality of coding. Such improvements in electronic documentation may benefit the quality of patient care, by ensuring that important clinical information is easily recalled.

### Conclusions

We developed a new algorithm, the Semi-Supervised Set Covering Machine, to identify clinical free text entries of interest. Our preliminary testing found that it worked effectively on free texts in the GPRD associated with two different medical conditions, and it may be of use in future research using electronic health records.

## Supporting Information

Figure S1 Pseudocode for S3CM algorithm.
(PDF)

Text S1 Description of Freetext Matching Algorithm.
(PDF)
