# Supplementary material for: Extracting Diagnoses and Investigation Results from Unstructured Text in Electronic Health Records by Semi-Supervised Machine Learning
Source: PLoS One. 2012 Jan 19;7(1):e30412. doi: 10.1371/journal.pone.0030412 (PMC3261909; doi:10.1371/journal.pone.0030412)
Supplement: Figure S1 — Pseudocode for S3CM algorithm. (PDF) [file pone.0030412.s001.pdf]

A. Original Set Covering Machine (SCM)

input: $\mathcal{P}$, $\mathcal{N}$, ρ, K

initialization: $f \leftarrow \emptyset$

repeat:

$\quad \hat{h} = \arg\max_{\substack{1 \le i \le n \\ h_i \notin f}} |\mathcal{P}(h_i)| - \rho \cdot |\mathcal{N}(h_i)|$

$\quad \mathcal{P} \leftarrow \mathcal{P} \backslash \mathcal{P}(\hat{h})$, $\mathcal{N} \leftarrow \mathcal{N} \backslash \mathcal{N}(\hat{h})$

$\quad f \leftarrow f \vee \hat{h}$

until $\mathcal{P} = \emptyset$ or $|f| \geq K$

return f

B. Modified SCM with feature generation

input: $\mathcal{P}, \mathcal{U}, \mathcal{N}$, ρ_1, ρ_2, K

$\quad f_{init}(\emptyset)$ *initial rule set* ($\emptyset$ *by default), used in bootstrapping*

initialization: $f \leftarrow f_{init}, \mathcal{U}_+ \leftarrow \emptyset$

$\quad \mathcal{H} \leftarrow \{h : h(x) \wedge h(x'), \exists x, x' \in \mathcal{P}, x \neq x'\}$

$\quad \mathcal{P}' \leftarrow \mathcal{P}, \mathcal{U}' \leftarrow \mathcal{U}, \mathcal{N}' \leftarrow \mathcal{N}$

repeat:

$\quad \hat{h} = \arg\max_{h \in \mathcal{H} \backslash f} |\mathcal{P}'(h)|$
$\quad\quad - \rho_1 \cdot |\mathcal{U}'(h)| - \rho_2 \cdot |\mathcal{N}'(h)|$

$\quad$ for each $x_+ \in \mathcal{P}'(\hat{h})$, $x \in \mathcal{U}'(\hat{h})$

$\quad\quad \mathcal{H} \leftarrow \mathcal{H} \cup \{h : h(x_+) \wedge h(x)\}$

$\quad$ end for

$\quad \mathcal{U}_+ \leftarrow \mathcal{U}'(\hat{h})$

$\quad \mathcal{P}' \leftarrow \mathcal{P}' \backslash \mathcal{P}'(\hat{h})$

$\quad \mathcal{U}' \leftarrow \mathcal{U}' \backslash \mathcal{U}'(\hat{h})$

$\quad \mathcal{N}' \leftarrow \mathcal{N}' \backslash \mathcal{N}'(\hat{h})$

$\quad f \leftarrow f \vee \hat{h}$

until $\mathcal{P} = \emptyset$ or $|f| \geq K$

return $f, \mathcal{U}_+$

C. Semi-supervised SCM

input: $\mathcal{P}, \mathcal{U}, \mathcal{N}$, ρ_1, ρ_2, K, M

initialization: $f \leftarrow \emptyset$, $\mathcal{P}_u \leftarrow \emptyset$, $t = 1$

for $i = 1$ to M

$\quad [f, \mathcal{U}_+] \leftarrow \text{mSCM}(\mathcal{P} \cup \mathcal{P}_u, \mathcal{U}, \mathcal{N}, \rho_1 t, \rho_2, K, f)$

$\quad \mathcal{P}_u \leftarrow \mathcal{P}_u \cup \mathcal{U}_+, \mathcal{U} \leftarrow \mathcal{U} \backslash \mathcal{U}_+$

$\quad t \leftarrow t + |\mathcal{U}_+|$

end for

return f, $\mathcal{P}_u$
