# Supplementary material for: Extracting Diagnoses and Investigation Results from Unstructured Text in Electronic Health Records by Semi-Supervised Machine Learning
Source: PLoS One. 2012 Jan 19;7(1):e30412. doi: 10.1371/journal.pone.0030412 (PMC3261909; doi:10.1371/journal.pone.0030412)
Supplement: Text S1 — Description of Freetext Matching Algorithm. (PDF) [file pone.0030412.s002.pdf]

Freetext algorithm documentation

Anoop Shah, Clinical Epidemiology Group

December 2011

This program extracts structured information in the form of Read terms, dates and numerical values from unstructured free text. This document describes the most updated version of the program. It is a Microsoft Access 2000 database but the algorithm itself does not use Access tables while working, except for data input and output.

The program was developed initially in 2003-2005 by Anoop Shah working for the General Practice Research Database (GPRD) Division of the Medicines and Healthcare products Regulatory Agency.

Contents

I. GENERAL DESCRIPTION AND USER GUIDE 7

1. Program output 7

2. Algorithm 7

2.1. Analysis modes 7
2.2. Rationale for design of the system 9
2.2.1. Selection of terms 11
2.2.2. Why we included OXMIS terms 11
2.2.3. Adding new codes 11
2.3. Analysis sequence 11
2.3.1. Sub main_termref 12
2.3.2. Sub main 13
2.4. Core tables 14
2.4.1. Terms table 14
2.4.2. Attrib2 table 15
2.4.3. Synonym table 15
2.4.4. Checkterms table 15

2.4.5. Ignore table 17
2.4.6. Ignore_phrase table 17
2.5. Machine-generated tables 18
2.5.1. Singlewords 18
2.5.2. Doublewords 18
2.5.3. Wordlist 18
2.6. Input and output tables 19
2.7. Maintenance 19
2.7.1. List of common English words: 2of4brif table 19
2.7.2. Read_attr1 table 21
2.7.3. Read_attr2 table 21
2.7.4. Oxmis_termref table 22

3. How to use the program **22**
3.1. Form freetext 22
3.2. Analysing a single text 22
3.3. Viewing the results 23
3.4. Analysing a set of texts 23
3.5. Importing texts to the input table 25
3.6. Using text files 25

4. Examples of analysis **25**
4.1. Normal mode 25
4.2. Append mode 25
4.3. Death mode ‘D’ 26
4.4. Lab test mode ‘L’ 26
4.5. Investigation result mode ‘N’ 26
4.6. Pregnancy mode ‘P’ 27
4.7. Sicknote mode ‘S’ 27
4.8. Time or date mode ‘T’ 27

5. Attributes **27**
5.1. Read terms 27
5.2. Dates 28
5.3. Duration 28
5.4. Lab tests 29

6. How to modify the algorithm tables **30**
6.1. Form terms2: terms 30
6.2. Form terms2: synonyms 30
6.2.1. Data entry fields 31
6.2.2. Buttons 31
6.2.3. How to add a new synonym 32
6.3. Form add_termlist 33
6.3.1. Set Include=TRUE for a single term 33

6.3.2. Set Include=TRUE for a set of terms . . . 33
6.3.3. Set the Read term category for a set of terms . . . 34
6.3.4. To regenerate the terms table . . . 34
6.4. Form attrib2 . . . 34
6.4.1. Buttons . . . 35
6.4.2. Format of attribute patterns . . . 36
6.4.3. Data entry fields . . . 37
6.4.4. How to add a new pattern for an existing attribute . . . 37
6.4.5. How to add a new attribute . . . 38
6.5. Other tables . . . 38

7. Testing the algorithm 38
7.1. Using the freetext form . . . 38
7.2. Analysis reports . . . 39
7.2.1. Overall analysis . . . 39
7.2.2. Readscore . . . 41
7.3. Aggregate reports . . . 42

8. Program development 43
8.1. Background . . . 43
8.2. Further development . . . 44
8.3. Major changes . . . 44
8.3.1. Information extracted . . . 44
8.3.2. User interface . . . 44
8.3.3. Changes made to increase speed . . . 44

II. GUIDE TO VISUAL BASIC CODE 45

9. Module freetext_core 45
9.1. Global variables and constants . . . 45
9.2. Sub init_all . . . 45
9.3. Function outfile_all As Boolean . . . 46
9.4. Sub main_termref . . . 46
9.5. Sub main . . . 47
9.6. Sub initial_search . . . 47
9.7. Sub attrib_search . . . 48
9.8. Sub analyse_pd . . . 48
9.9. Function remove_ignorable As String . . . 49
9.10. Function readscore As Single . . . 49
9.11. Function fuzzylink As Long . . . 50

10. Module attrib 50
10.1. Global variables and constants . . . 50
10.2. Sub init . . . 50

10.3. Sub infile 50
10.4. Sub outfile 51
10.5. Function dissect2_options As String 51
10.6. Sub pd_search2 51

11. Module checkterms **51**
11.1. Global variables and constants 52
11.2. Sub init 52
11.3. Sub infile 52
11.4. Sub outfile 52
11.5. Sub check_all 52
11.6. Function in_list As Long 53
11.7. Function if_qualify As Boolean 53
11.8. Function if_dequalify As Boolean 53

12. Module list **54**
12.1. User-defined data types 54
12.2. Global variables and constants 54
12.3. Function bestmatch As String 54
12.4. Function expand As termlist 54
12.5. Sub test 55
12.6. Sub display 55
12.7. Function add_termlists As termlist 55
12.8. Function getlist As termlist 56
12.9. Function wordtermrefs_count As Long 56

13. Module pd **56**
13.1. Global variables and constants 57
13.2. Sub check_compressed 57
13.3. Sub remove_from_compressed 57
13.4. Sub compress 58
13.5. Function correct_attr As Boolean 58
13.6. Sub show_all 58
13.7. Sub show_all_2 58
13.8. Function true_ As Long) 59
13.9. Function Attr As String 59
13.10Function mean As String 59
13.11Sub del_attr 60
13.12Sub set_attr 60
13.13Sub set_mean 60
13.14Sub add_attr 60
13.15Sub add_mean 61
13.16Function part_nopunc As String 61
13.17Function part_punc_nospace As String 61
13.18Function matchpattern As Boolean 62

13.19 Function matchposition As Boolean 62
13.20 Function matchoption As Boolean 62
13.21 Sub init_read 63
13.22 Function st_type As Long 63
13.23 Sub clear 63
13.24 Sub remove 63
13.25 Function text As String 64
13.26 Sub set_text 64
13.27 Function punct As String 64
13.28 Function max As Long 64
13.29 Function part_punc As String 65

14. Module strfunc **65**
14.1. Global variables and constants 65
14.2. Function get_date As String 65
14.3. Function get_date_average As String 66
14.4. Function words As String 66
14.5. Function in_set As Boolean 66
14.6. Function is_text As Boolean 67
14.7. Function numwords As Long 67
14.8. Function is_acronym As Boolean 68
14.9. Function all_punc As Boolean 68
14.10 Function matchindex As Single 68
14.11 Function num_diff_char As Long 68
14.12 Function dissect As String 69
14.13 Function dissect2 As String 69
14.14 Function is_numeric As Boolean 70
14.15 Function phrase_match_pattern As Long 70

15. Module synonym **70**
15.1. Global variables and constants 71
15.2. Sub infile 71
15.3. Sub outfile 71
15.4. Sub init 71
15.5. Sub del 72
15.6. Sub add 72
15.7. Sub add_with_acronym 72
15.8. Function get_search_summary As String 72
15.9. Function trylink_2 As String 73
15.10 Function s2_pos As Long 73
15.11 Function s1_pos As Long 73
15.12 Function s2 As String 74
15.13 Function s1 As String 74
15.14 Function s1_priority As Long 74

16. Module terms **74**
16.1. Global variables and constants 74
16.2. Sub infile 75
16.3. Sub outfile 75
16.4. Sub init 75
16.5. Function true_term As Boolean 75
16.6. Function exact_read_termref As Long 75
16.7. Function read_type As String 76
16.8. Function std_term As String 76
16.9. Function attrib_str As String 76

17. Module wordlist **76**
17.1. Global variables and constants 77
17.2. Sub infile 77
17.3. Sub outfile 77
17.4. Sub init 78
17.5. Sub init_ignore 78
17.6. Function in_wordlist As String 78
17.7. Function in_wordlist_OLD As Boolean 78
17.8. Function approx_wordlist As Long 79
17.9. Function pos_wordlist As Long 79
17.10Function pos_singlewords As Long 79
17.11Function pos_doublewords As Long 79
17.12Function sng_termref As Long 80
17.13Function dbl_termref As Long 80
17.14Function dbl_numwd As Long 80
17.15Function wordlist_termref As Long 80
17.16Function termref_in_singlewords As Boolean 81
17.17Function termref_in_doublewords1 As Boolean 81
17.18Function termref_in_doublewords2 As Boolean 81
17.19Function wordsearch As String 81
17.20Function ignorable As Boolean 82
17.21Function ignore_max As ignore_max() 82
17.22Function ignore_words As String 82
17.23Function remove_ignore_phrases As String 82
17.24Function initial_process As String 83

18. Module in_out **83**
18.1. Function filepath As filepath() 83
18.2. Function input_string As String 83
18.3. Function read_term As String 83
18.4. Sub import_input_table 84
18.5. Sub do_input_table 84
18.6. Sub results_output 84
18.7. Sub do_text_file 85

19. Module maintenance **86**
19.1. Global variables and constants 86
19.2. Sub init_read_attr_tables 86
19.3. Function read_attribute As String 86
19.4. Function make_std_term As String 87
19.5. Function make_std_term_with_attr As String 87
19.6. Sub rm_attr 87
19.7. Sub delete_long_terms 88
19.8. Sub delete_superfluous_terms 88
19.9. Function find_similar_term As Long 88
19.10Sub make_wordlist 88
19.11Function count_t As Integer 89
19.12Sub term_remove_BATCH 89
19.13Sub term_remove_NEW 89
19.14Function read_code_oxmis As String 89
19.15Sub process_termlist 90
19.16Sub expand_rightleft 90

Part I. GENERAL DESCRIPTION AND USER GUIDE

1. Program output

The program processes unstructured free text and produces a table of structured data elements (data type, attribute, value), as shown in Table 1.

2. Algorithm

The program is designed to extract diagnostic Read terms and several other types of structured data from unstructured free text. In order to do so it uses the table of Read and OXMIS terms, as well as several custom tables for detection of patterns and conversion of synonyms.

2.1. Analysis modes

The analysis mode can be selected automatically based on the meaning of the Read term associated with the text.

Data type	***Possible attributes***	***Value***	***Description***
**`READ`**	*Death mode:* **`Deathcause, deathcause1a, deathcause2`** etc. *Others:* **`Family, negfamily, query`** + others	**`Termref_uid`** of matched Read term	Match to Read diagnostic term (i.e. lettered chapter). Tests, family history, personal history, investigations and administrative terms are currently not used.
**`DATE_full, DATE_year, DATE_time`**	*Death mode:* **`deathdate, certdate`** *Others:* **`admitdate, followup, dob, edd, lmp, dateprev, datenext`**	Date	Date in various formats.
**`DURA_yrs_, DURA_mths, DURA_wks_, DURA_days`**	**`duraprev, duranext, followup, age`**	Number	Duration in various formats.
**`LABS`**	**`gest, sysbp, diabp, haemoglobin, mcv, pulse`** and others	Numerical value or ‘normal’, ‘abnormal’, ‘low’, ‘high’	Laboratory values. ‘gest’ is gestational age in weeks.
**`ATTR`**	**`family, negative, query`**	none	If the text suggests that the Read Term does not refer to a definite diagnosis for this patient (e.g. “*Death* of mother”, “*Pneumonia* possible”)

Table 1: Structured data format

Death searches for the cause of death and interprets 1a, 1b etc. as death certificate entries. Laboratory results are not extracted.

Pregnancy a duration given in weeks is interpreted as gestational age if it is less than 43 weeks.

Labtest a numerical value or 'normal', 'abnormal' etc. can be interpreted as the test result

Normal / abnormal 'normal', 'abnormal', 'nad', 'positive' etc. can be interpreted as the investigation result, but numerical values cannot.

Date only a single date is allowed in the output.

Sicknote dates are regarded as medical certificate start or end dates.

2.2. Rationale for design of the system

The UK General Practice Research Database (GPRD) is a large database of primary care records and is an important source of clinical information for epidemiology and drug safety research. It contains details of consultations, diagnoses, test results, prescriptions and referrals. General practitioners (GPs) code important diagnoses using a structured clinical terminology. Currently the 'Read' clinical terminology is used, but OXMIS (Oxford Medical Information System) was used previously, and SNOMED-CT (Systematized Nomenclature of Medicine–Clinical Terms) will be used in the future. Additional information is entered in free text associated with the coded entries.

Our aim was to develop a natural language processing system to extract diagnoses as Read terms from free text in the GPRD, thus allowing researchers to combine information in coded and unstructured data in research studies using primary care data. We chose to develop our system independently rather than adapting an existing system so that we would have access to all the code and would be able to optimise its performance. Our eventual aim is to develop software suitable for use by doctors when entering data. This will enable the majority of clinical information to be coded at the time of data entry, with minimal cost on the doctor's time.

Our 'Freetext' program uses manually entered lookup tables of phrases and synonyms, and simple semantic information from the Read terms themselves (e.g. negation) to identify appropriate Read terms for diagnoses stated in the text. The Read and OXMIS dictionaries were designed for coding by GPs and already incorporate synonymous terms with variations in the way doctors may express common diagnoses. We manually created a synonym table to allow the program to interpret a greater range of alternative phrases.

The algorithm was developed by an iterative process. After writing the initial program, we used it to analyse samples of several hundred randomly chosen free text entries, and reviewed the output manually. As well as the final structured output, the program produced a detailed report of the intermediate stages of analysis to make it easier to find the root cause of any mistake. We modified the lookup tables and program code based on the results of each test, re-tested the program on the same sample to verify that the errors had been resolved, and then tested it on a new sample of texts.

Clinical terminology

The algorithm was principally designed to encode diagnoses in the free text to terms in the Read Clinical Terminology, which is the system used for the existing coded entries in the GPRD. Apart from diagnoses, the Read terminology includes codes for other categories of information such as history, examination findings, procedures and test results. Our algorithm was designed to extract some of these entries but the main focus was on diagnoses.

When GPRDÂăstarted collecting data from general practice computer systems in 1987, practices were using the OXMIS dictionary (OXford Medical Information System) to encode clinical entries. Practices switched over to the Read dictionary at varying dates in the 1990s. The current GPRD (in 2011) uses only Read terms (with OXMIS terms having been converted to the Read equivalents), but when we started developing the software in December 2003, GPRD contained a mixture of Read and OXMIS terms, with a total of 104,802 terms available.

We standardised the wording of Read terms by replacing abbreviations such as ‘a/n’ (antenatal) with the full word, and removing phrases such as ‘NEC’ (not elsewhere classified) which would not be found in ordinary clinical text; the list of such replacements is in database table Read_Attr1 (subsubsection 2.7.2). If some Read terms became identical after this process of standardisation, only one of them was retained. We categorised each word in a Read term as positive, negative, optional or ignorable, using the Read_Attr2 table (subsubsection 2.7.3). For example, for the Read term K510000 ‘Cystocele without uterine prolapse’, the word ‘cystocele’ would have to have a positive attribute, ‘uterine prolapse’ would have a negative attribute and ‘without’ would be ignored. We used these allocations to define which words in a Read term need to be present in the text in order for the term to be matched. For example, in order to match the Read term B723z00 ‘Benign neoplasm of bronchus or lung NOS’, a phrase would only need to include one of the words ‘bronchus’ or ‘lung’. Short words which would not alter the meaning of the term if omitted (e.g. ‘of’, ‘or’ and ‘NOS’ in the example), and words which influence the true / false status of nearby words but have no other meaning (e.g. ‘lack of’) were designated as ignorable and did not need to be present in the text.

We wrote a function called ‘readscore’ to grade the closeness of a match between a text phrase and a Read term, with a minimum threshold for a satisfactory match (see subsubsection 7.2.2 and subsection 9.10). The function attempts to map each word or phrase in the text to a word or phrase in the Read term, and vice versa, in order to verify that they have the same meaning. Points are deducted from the readscore for each ignorable or negative word not matched, and for use of synonyms rather than identical words. The algorithm tries a number of possible matches for a text phrase and choose the match with the highest readscore. For example, the text phrase ‘Benign neoplasm of lung’ would preferentially be matched to the Read term with the same wording, even though it could also match ‘Benign neoplasm of bronchus or lung NOS’ with a lower readscore. However, for the text phrase ‘Benign neoplasm of bronchus’, there is no specific term, so the only valid match would be with the Read term ‘Benign neoplasm of bronchus or lung NOS’.

2.2.1. Selection of terms

We manually defined a subset of terms which the algorithm was allowed to select. Terms with more than 5 non-ignorable words were excluded as they are too long and complex to match and are infrequently used. The final list contained 42,931 terms which the algorithm was allowed to select. Of these, 38,981 encoded medical diagnoses. This is particularly the case for Read Chapter 'T', which contains over 3000 terms describing specific (and often rare) external causes of injury, e.g. 'T546000 Sucked into jet - occupant of spacecraft injured'.

2.2.2. Why we included OXMIS terms

The OXMIS dictionary is no longer used by GPs to encode information, and these codes have been replaced by their Read equivalents in most recent version of GPRD. However, we retained OXMIS codes in our program because they provide additional ways of expressing common diagnoses, and in some cases the mapped Read term is not exactly equivalent. For example, Read contains the term K510000 'Cystocele without uterine prolapse', but there is no Read term for cystocele without a statement of uterine prolapse. However, OXMIS contains the lone term 'CYSTOCELE', and this would be the preferred match to an unqualified statement about cystocele in the text. More than one OXMISÂăterm may map onto a single Read term, thus some precision is lost with code conversion. We therefore retained OXMIS terms in the algorithm, but the output can easily be converted to Read terms if required.

2.2.3. Adding new codes

Our system was designed to enable the easy addition of new codes, which may be useful for coding emerging diseases even before they are recognised in official coding terminologies. To demonstrate this concept, we created the terms 'Recently in hospital' and 'Rhabdomyolysis' because they were not included in Read or OXMIS, but can encode clinically useful information which may be present in free text.

2.3. Analysis sequence

Figure 1 gives an overview of the analysis sequence, which is described in more detail in the following sections.

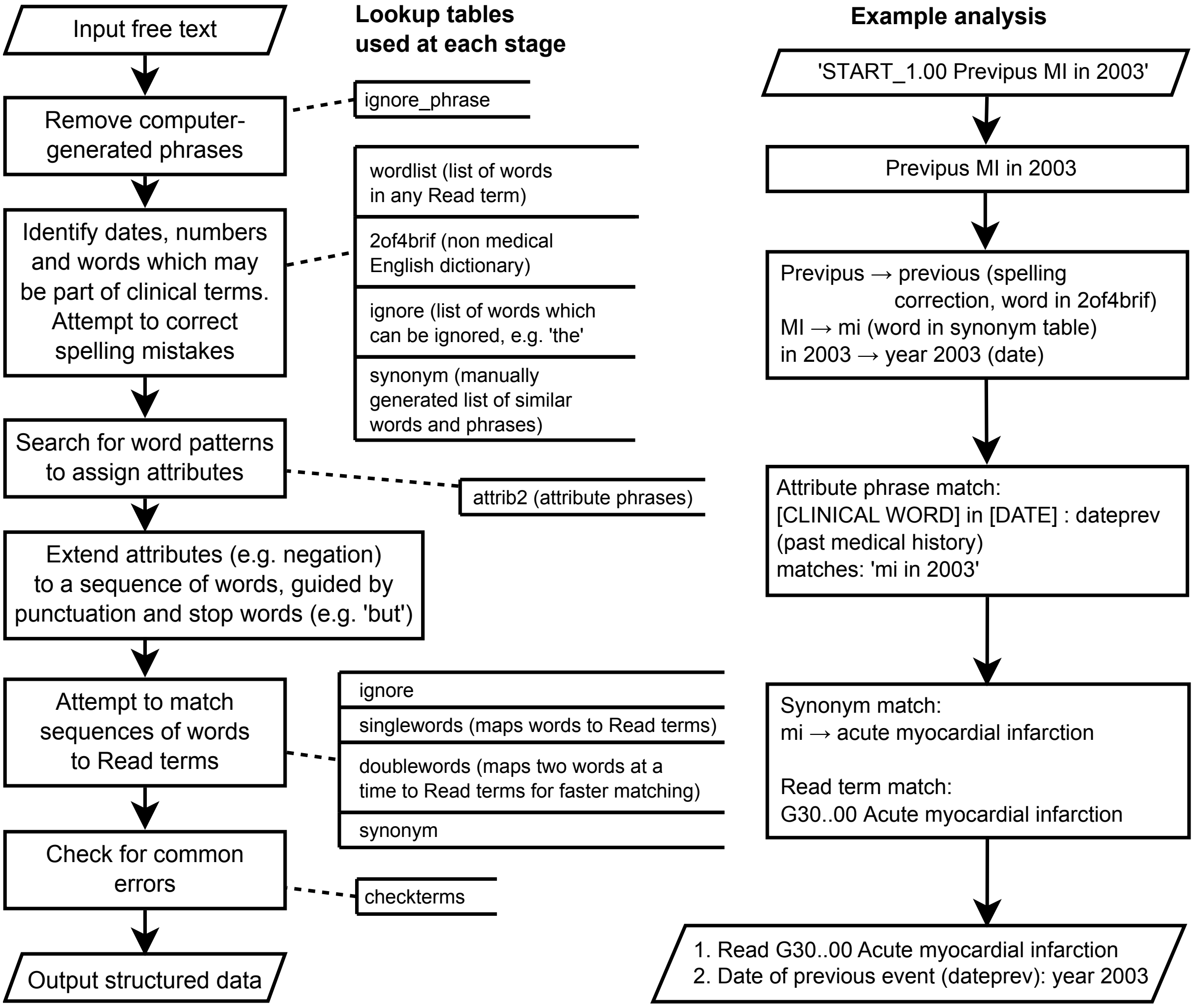

Figure 1: Overview of the steps involved in analysing a text, showing the lookup tables used at each stage and an example analysis

2.3.1. Sub main_termref

This Sub calls **main** (see below) with the appropriate analysis options based on the termref of the original Read term.

One of the options is **append**, which can be set to TRUE if the free text should be appended to the Read term text (to appear as it would on the doctor's computer). Text is not appended if the Read term type is LABS, DATE or SICKNOTE. The Read term is analysed separately, and the first interpreted value from the main text is removed if it is the same as that from the Read term alone or the existing termref and there is no attribute. *MYOCARDIAL INFARCT* "Anterolateral" →

(no output)

MYOCARDIAL INFARCT "Father" →

1 READ family 303768 MYOCARDIAL INFARCT

See subsection 9.4 on page 46 for technical details.

2.3.2. Sub main

Carries out the analysis of **instring** according to the analysis options. The results are stored in the arrays in module **pd** (see section 13 on page 56). If **debug_** is TRUE, the intermediate processes are documented in the global variable **debug_string** (see page 39). See subsection 9.4 on page 46 for technical details. The analysis sequence is as follows (see Figure 1):

1. Initialise the **readscore** function (because it stores previous results); (subsection 9.10 on page 49)
2. Meaningless computer-generated phrases (e.g. "Hide=N") are removed from the free text by **remove_ignore_phrases** (see subsection 17.23 and subsubsection 2.4.6). If the text appears to be from a structured data area, the function **wordlist.initial_process** (subsection 17.24) extracts the useful information and converts it to a form suitable for further analysis.
3. The words and punctuation are assigned to arrays (module **pd**; by Sub **pd.init_read**; see subsection 13.21.
4. The array of words is searched for dates, numbers, and entries in the synonym, ignore and wordlist tables. A data type is assigned to each word, which is **CLIN** (i.e. may be part of a Read term) for words in the synonym and wordlist tables. This search is carried out by (Sub **initial_search**; see subsection 9.6.
5. The procedure **attrib.pd_search2** uses the **attrib2** list (described on page 15) to find matching patterns, and attributes are assigned to phrases which match (see subsection 10.6).
6. The attributes are extended to nearby words according to certain rules in the program (e.g. a **negative** attribute is continued until a full stop, colon or the word 'but' is reached).
7. The text is analysed in sequences of up to 5 words with the data type **CLIN**. The words may be adjacent or may have 'ignore' words in between, such as 'the', 'and' etc. This is carried out by Sub **attrib_search** (see subsection 9.7 on page 48).
8. Call codeanalyse_pd to attempt to match sequences of clinical words to Read terms (subsection 9.8 on page 48). The program tries to find a Read term which matches the largest possible number of adjacent words, using the synonym table to link alternative phrases with the same meaning. Each word or phrase in the text is mapped to an individual word or phrase in the Read term, so the order of words does not matter. Negative parts of the text must map to negative parts of the Read term.
9. Call **pd.compress** to filter to include only the structured data extracted (subsection 13.4 on page 58).
10. Call **pd.check_compressed** to check that each structured data element has an appropriate attribute and value for its data type (subsection 13.2 on page 57).

11. The output is condensed into a sequence of converted dates and Read terms with associated attributes. In some cases the output consists solely of an attribute, e.g. if the text consists of "father" then the output is `ATTR family` without a Read term.

12. Call `checkterms.check_all` for a final validity check for a selected set of Read terms (subsection 11.5 on page 52).

2.4. Core tables

2.4.1. Terms table

A list of all Read and OXMIS terms used in the GPRD. It will need to be updated when new terms are used in the future. This table is used to derive the appropriate analysis mode and to test the text against candidate Read terms for conversion. Fields:

Termref GPRD term reference Uid

Code Read or OXMIS code

Term Original text version of term

Readcode For Read terms, this is the same as `Code`. For OXMIS codes, it is the Read code of the corresponding Read term. It enables the use of the Read hierarchy for term viewing and selection.

chap1 First character of `Readcode`, for fast filtering by the `terms2` form (see page 30).

chap2, chap3 Second and third characters of `Readcode`

Type Read term type:

D death

L investigation result which may have a numerical value

M medical (diagnostic) term (i.e. Read lettered chapter)

N investigation result which can only have a non-numerical value

P pregnancy

T time or date

S sick note

Attr Code describing whether each word of the Read term is true, false or ignorable (see page 30)

std_term standardised version of the Read term

Read TRUE if it is a Read term, FALSE if it is OXMIS.

Include TRUE means that the terms is used for text conversion output. This includes most of the Read lettered chapters and selected terms from immunisations, symptoms, laboratory

results and physical examination. Many terms have been set to `Include`=FALSE, either manually if they are ambiguous, or automatically if they are too long.

2.4.2. Attrib2 table

Context patterns. Modified using form **attrib2** (page 34). Fields:

w1, w2, w3, w4, w5 up to 5 words in the pattern. See Table 4 on page 36 for details.

p1, p2, p3, p4, p5 punctuation following the 5 words.

a1, a2, a3, a4, a5 attribute to assign.

order search order. Lower numbered patterns can overwrite the changes made by higher numbered patterns.

death_only TRUE if this pattern is only used in ‘Death’ mode.

text for reference only; not used by program

date date pattern entered.

2.4.3. Synonym table

A manually generated list of similar words and phrases, modified using form **Terms2** (page 30). Fields:

s1 word or phrase (up to 5 words) which might occur in free text

s1_num number of words in `s1`

s2 word or phrase (up to 5 words) which might occur in Read term

s2_num number of words in `s2`

priority number coding the accuracy of the link (see page 32)

date date entry made

2.4.4. Checkterms table

This table lists ‘qualifying’ and ‘dequalifying’ strings for some termrefs. It has the following fields:

termref termref (link to `terms` table)

qualify comma separated list of qualifying words

dequalify comma separated list of dequalifying words

Some Read terms may appear in the text but may not apply to the current patient, according to the context. For example, “malaria” in the context of “prophylaxis” should not be converted to the Read term for malaria, or should have an attribute which shows that it is not a current diagnosis for this patient. The current version of the program does not have a specific attribute for ‘prophylaxis’, so in this case the word should simply be ignored.

Another example is “AF”, which usually means “atrial fibrillation”, but could also mean “anterior fontanelle” or something else. The program would convert this to ‘atrial fibrillation’ using the synonym table, which would be encoded as **`READ 261645`** (Atrial fibrillation). This termref is allowed only if the original text or Read term contains “ischaemic”, “heart”, “hypertens” etc. so that other instances of “AF” are not erroneously converted.

If a term has ‘qualifying’ words, then one of those words must be present in the original Read term or associated free text in order for the converted term to be allowed. If a qualifying word is not present, the Read term is removed from the output.

If a term has ‘dequalifying’ words, it is removed from the output if any of the dequalifying words is present. If both a qualifying and dequalifying word is present, the output is marked with the attribute **`machinequery`**, which marks it as requiring manual checking.

The table is edited using the form **`checkterms`** (see Figure 2).

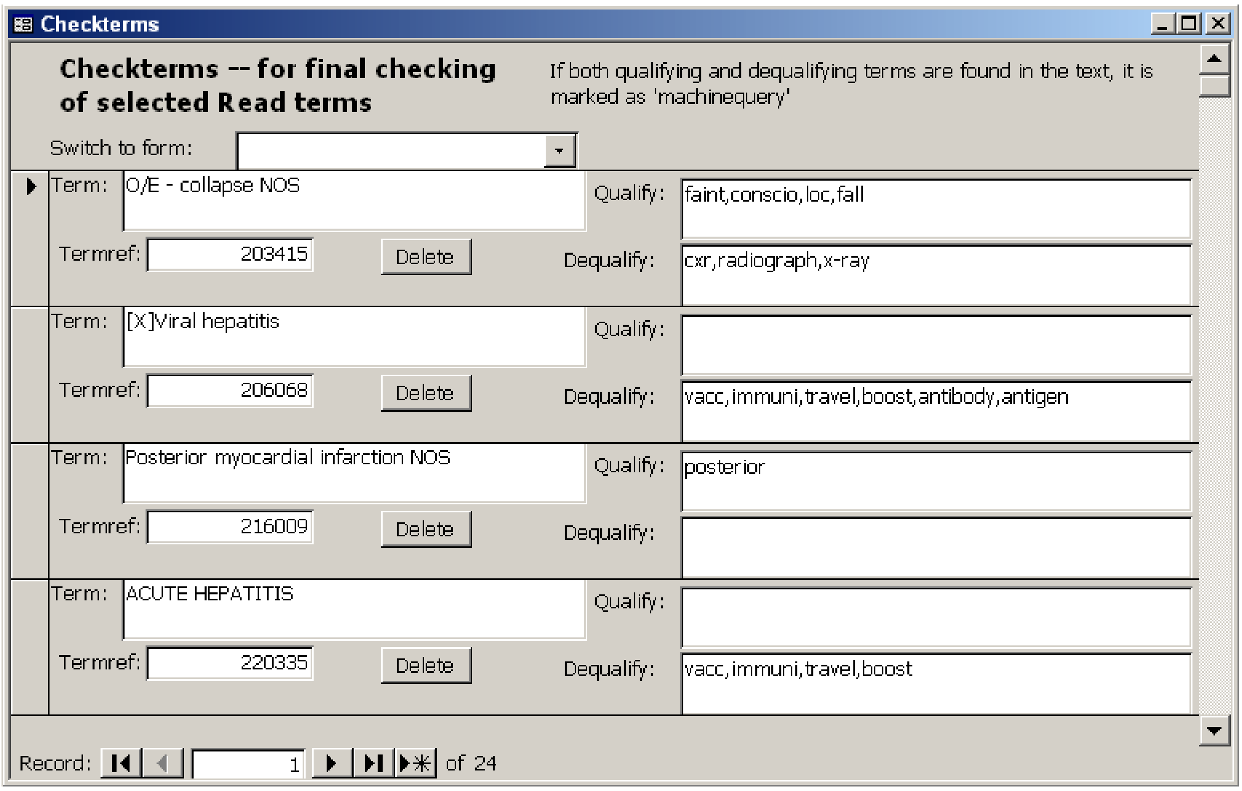

Figure 2: Checkterms form

2.4.5. Ignore table

This table consists of a single field (**word**) which lists ‘ignorable’ words and phrases. These are words which frequently occur in medical notes but do not relay any important information. For example, the word ‘of’ is ignorable to enable ‘injury of knee’ to map to ‘knee injury’. This table can be edited using the **ignore** form (see Figure 3).

Examples: ‘and’, ‘as’, ‘at’, ‘became’, ‘becoming’, ‘by’, ‘caused’, ‘causing’, ‘complains of’

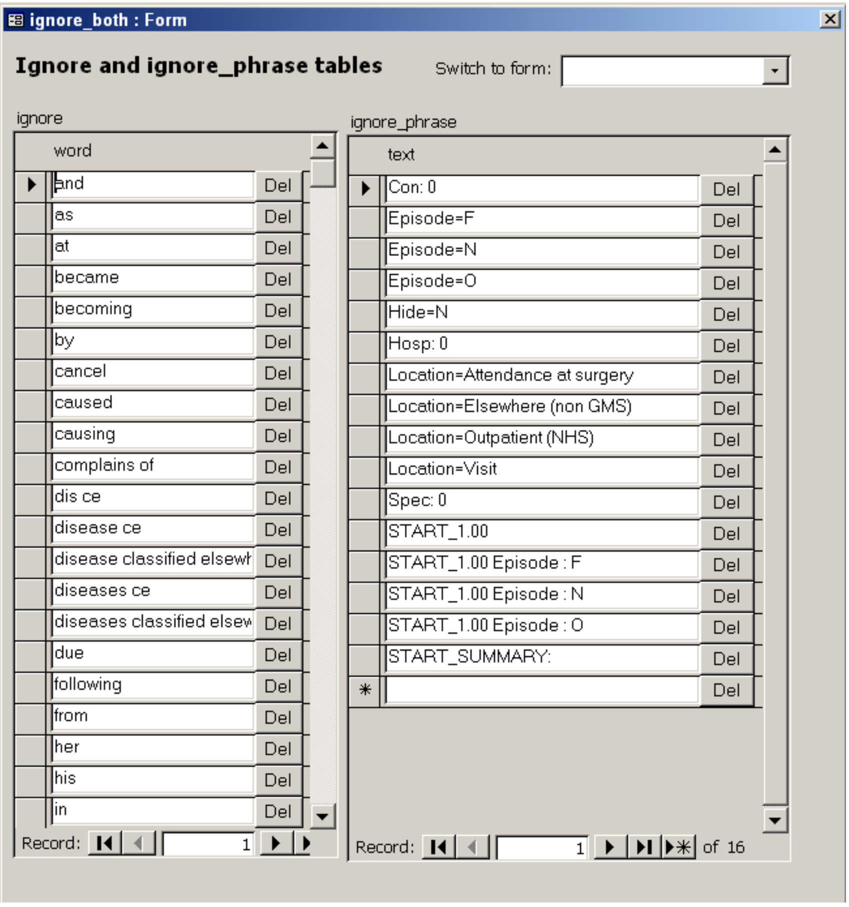

Figure 3: Form for editing the ignore and ignore_phrase tables

2.4.6. Ignore_phrase table

This table also has just one field (**text**) which lists *exact* phrases (including specific spacing and correct case) which are to be ignored because they are part of a structured data format. This format may be specific to VM or Vision; modifications to this table may be required if the program is to be used with other data sources. Words or phrases can be preceded by **START_** to indicate that they apply only at the beginning of the free text string.

Examples:

Text
Con: 0
Episode=O
Hide=N
Location=Elsewhere (non GMS)
START_1.00
START_1.00 Episode : O
START_SUMMARY:

2.5. Machine-generated tables

These tables are generated by sub **`make_wordlist()`** in module **`maintenance`**. They are generated from the **`std_term`** of the subset of Read terms for which **`Include`**=TRUE.

2.5.1. Singlewords

Each termref contributes one row per non-ignorable word. The table has 3 columns:

words a single word which appears in a Read **`std_term`**

termref the termref in which the word appears

numwd the number of non-ignorable words in the **`std_term`**

2.5.2. Doublewords

This table is similar to **`singlewords`** except that the **`words`** column contains every pair of non-ignorable words that appears in a Read term, with each pair in alphabetical order. For example, Read term 279879 'Infective otitis externa due to erysipelas' would generate the following rows:

**termref**	**numwd**	**words**
279879	4	infective otitis
279879	4	externa otitis
279879	4	externa infective
279879	4	erysipelas externa
279879	4	erysipelas otitis
279879	4	erysipelas infective

2.5.3. Wordlist

This table has 2 columns:

words every word which occurs in a **`std_term`**

wordlength the number of letters in the word

It is used in conjunction with the **2of4brif** table (see subsubsection 2.7.1 on page 19). The actual lookup table is generated by the union of the two tables:

```
SELECT wordlist.words, wordlist.wordlength, TRUE As Clinical FROM
wordlist UNION
SELECT [2of4brif].words, [2of4brif].wordlength, FALSE FROM [2of4brif]
ORDER BY wordlength ASC, words ASC, Clinical ASC;
```

The list is sorted by **wordlength** and then by **words** alphabetically, enabling the program to search for a particular word and quickly find similar words of the same length.

2.6. Input and output tables

The database contains tables which can be used to analyse a batch of texts and check the results.

Input Text files can be imported into this table, and it also stores individual texts added separately. See Table 2.

Output Output from analysis. See Table 3.

Debug This table stores the analysis reports generated from analysis of each individual text if the appropriate option is selected (see subsection 7.2 on page 39).

Reports A list of SQLs, descriptions and accuracy results based on manual checking of the program output. The queries can be modified and run from the form **reports** (see subsection 7.3 on page 42). Fields: **ID, description, sqltext, result**

File_location Fields **file type** and **path**. The path for 'Data' is the default entry in the data entry fields of form **batch** (see subsection 3.4 on page 23). The path for 'Wordlist' is for a temporary text file used by the program for storing lookup tables.

2.7. Maintenance

2.7.1. List of common English words: 2of4brif table

This is a publicly available list of common English words downloaded from http://wordlist.sourceforge.net/.
Documentation is provided at wordlist.sourceforge.net/12dicts-readme.html. This list is used in conjunction with **wordlist** for the spell checker, to ensure that common non-medical words are not misinterpreted as mis-spelt medical words. The two fields are **words** and **wordlength**, which correspond to the fields in the **wordlist** table (see subsubsection 2.5.3 on page 18).

Field name	***Data type***	***Description***
id	Integer	text_uid or automatic id
text	rrMemo (long string)	free text to be interpreted
termref	Integer	associated Read term
checked	Boolean	whether manually checked
read_missed	Integer	number of Read diagnoses not detected
dates_missed	Integer	number of dates with attributes not detected
labs_missed	Integer	number of lab results not detected
comments	Text	comments entered during checking
term_extra	Boolean	whether the converted term is more detailed than the original term
term_corr_happ	Boolean	whether analysis of the free text correctly shows that the original Read term does not apply to this patient

Table 2: Input table fields

Field name	***Data type***	***Description***
id	Integer	links to id column of `input` table
order	Integer	order of output row from a single text
type	Text	data type (see Table 1)
attribute	Text	attribute (see section 5)
value	Text	termref, date or lab value
extra	Text	Read term, if data type is `READ`
auto_happ	Boolean	whether the algorithm considers that this Read term is an event which happened to this patient (i.e. attribute not ‘family’, ‘negpmh’, ‘negative’ or ‘query’). Positive for all dates with attribute and all lab results.
actual_happ	Boolean	whether Read term is correct and *actually* happened to this patient (based on manual review), or whether the date or lab result is correct and applies to this patient
important	Boolean	can be manually set to FALSE if the term is a duplicate; otherwise TRUE. If set to FALSE, the term is not considered in calculations of sensitivity or specificity.
corr_attr	Boolean	whether the attribute is optimal
corr_value	Boolean	whether the Read term is optimal

Table 3: Output table fields

2.7.2. Read_attr1 table

This table gives exact phrases or patterns in Read terms which may need to be ignored or replaced in order to generate the **std_term**. Phrases can be removed or replaced (e.g. 'a/n' by 'antenatal'), and the patterns can apply only at the start or end of the Read term if necessary. Part of the table is reproduced below.

Raw_pattern	Replacement	Order	Position	Comment
[x]		4	START	ICD-10 codes
[so]		5	START	Site of operation
other		6	START	
nos		7	END	
nec		8	END	
symptom		9	END	
not otherwise specified		10	END	
not elsewhere classified		11	END	
site not specified		12	END	
a/n	antenatal	13		
h/o	history of	14		

The Read_attr1 and read_attr2 tables can be modified using the form **read_attr**. Both tables are sorted by the **Order** column which must contain unique integers. The search order can be altered by rearranging these numbers.

2.7.3. Read_attr2 table

This table consists of a list of rules for producing the Read attribute string. This denotes whether each word in the **std_term** version of the Read term is True (**T**), False (**F**), ignorable (**I**) or is an option (**O**) (e.g. 'Retained placenta or membranes' = **TOIO**). The following special codes can be used: **#**=number, *=text, **?**=anything. Punctuation is treated as a word at this stage but is coded **P**; these letters are removed from the final attribute string because the **std_term** to which it refers does not have any punctuation. Here are some examples from this table:

Pattern	Attribute	Order	Comment
* or *	OIO	1	
* / * / *	OIOIO	2	
* / *	OIO	3	
* # / * #	TTITT	11	e.g. c6/c7
requires a *	TIT	12	
not yet *	IIF	13	
not yet * *	IIFF	14	
not signifying *	IIF	40	
, * neg	PFI	51	
not specific * *	IIFF	52	
not specif * *	IIFF	53	
without *	IF	54	

2.7.4. Oxmis_termref table

This table lists the termrefs of OXMIS terms (field: `termref`) and the Read terms to which they map (field: `read_termref`). More than one OXMIS term can map to a single Read term.

3. How to use the program

Open the form `freetext` (see Figure 4). This displays one text at a time with the results of the analysis. There is a window which displays an analysis report, which can be used to diagnose the problem if there is an error in the analysis. The error can be prevented in the future by modifying the terms, attributes or synonym tables, which are accessible via buttons on the freetext form.

3.1. Form freetext

This form cycles through the rows in the input table. The analysis report is stored in the debug table, and the output subform is based on the output table. The associated Read term is obtained by linking to the terms table.

3.2. Analysing a single text

Click Test new phrase. This will bring up a dialog box in which the phrase can be pasted or typed. Further dialog boxes will be brought up for analysis options:

- whether to use spelling correction
- the termref or type of the associated Read term

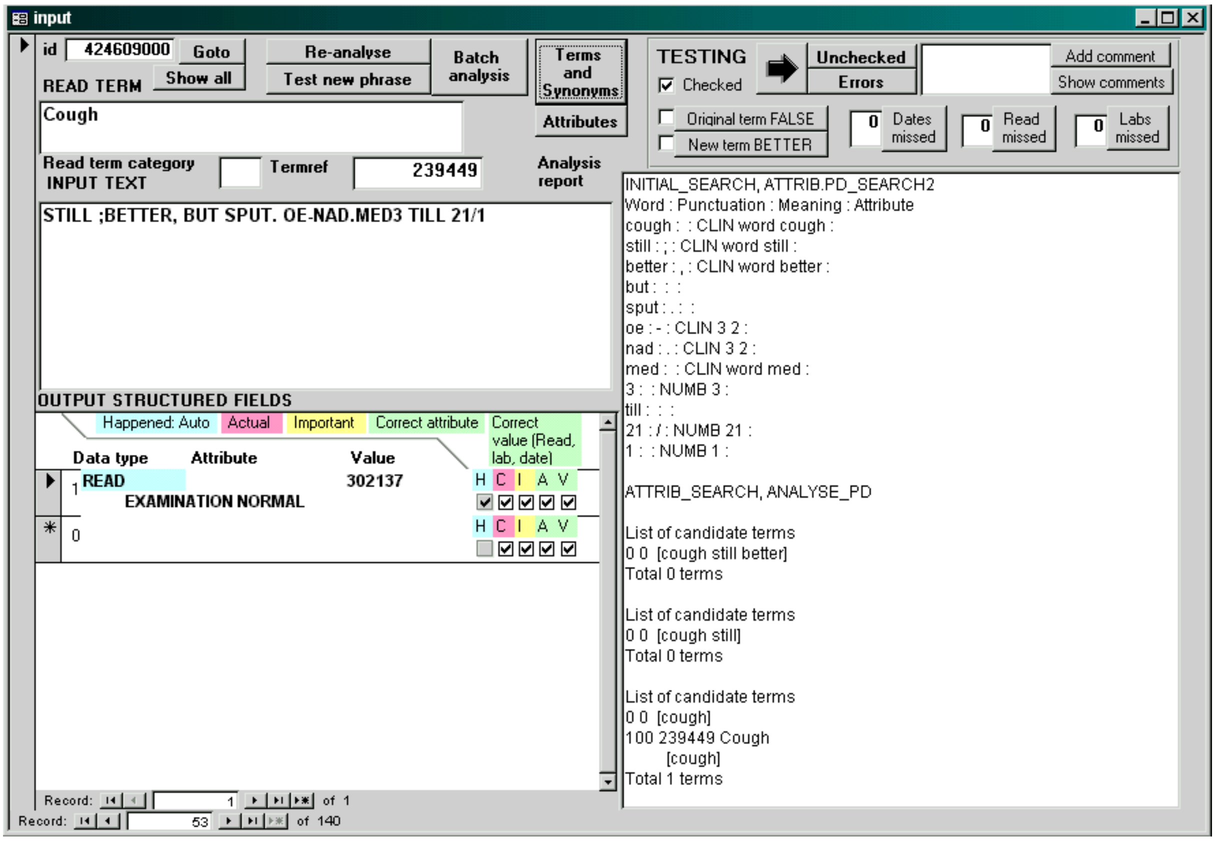

Figure 4: Freetext form

- whether to append the free text on the end of the Read term and analyse both together

The text and the analysis results are added to the **input** and **output** tables. The analysis report is added to the **debug** table, and displayed to the right of the freetext screen. To re-analyse the current text, click Re-analyse current phrase.

3.3. Viewing the results

- Click Goto in the top left-hand corner to go to a specific ID.
- Click Show all to make the form cycle through all texts (default).
- Click Show unckecked to cycle through all texts with ‘checked’=FALSE.

3.4. Analysing a set of texts

Open the form **batch** (this can be done by clicking Batch analysis on the form **freetext**); see Figure 5.

There are two options for analysing a set of texts: either importing them to the input table and placing the structured output in the output table, or using text files. The former option allows the form **freetext** to be used to inspect the analysis results one text at a time.

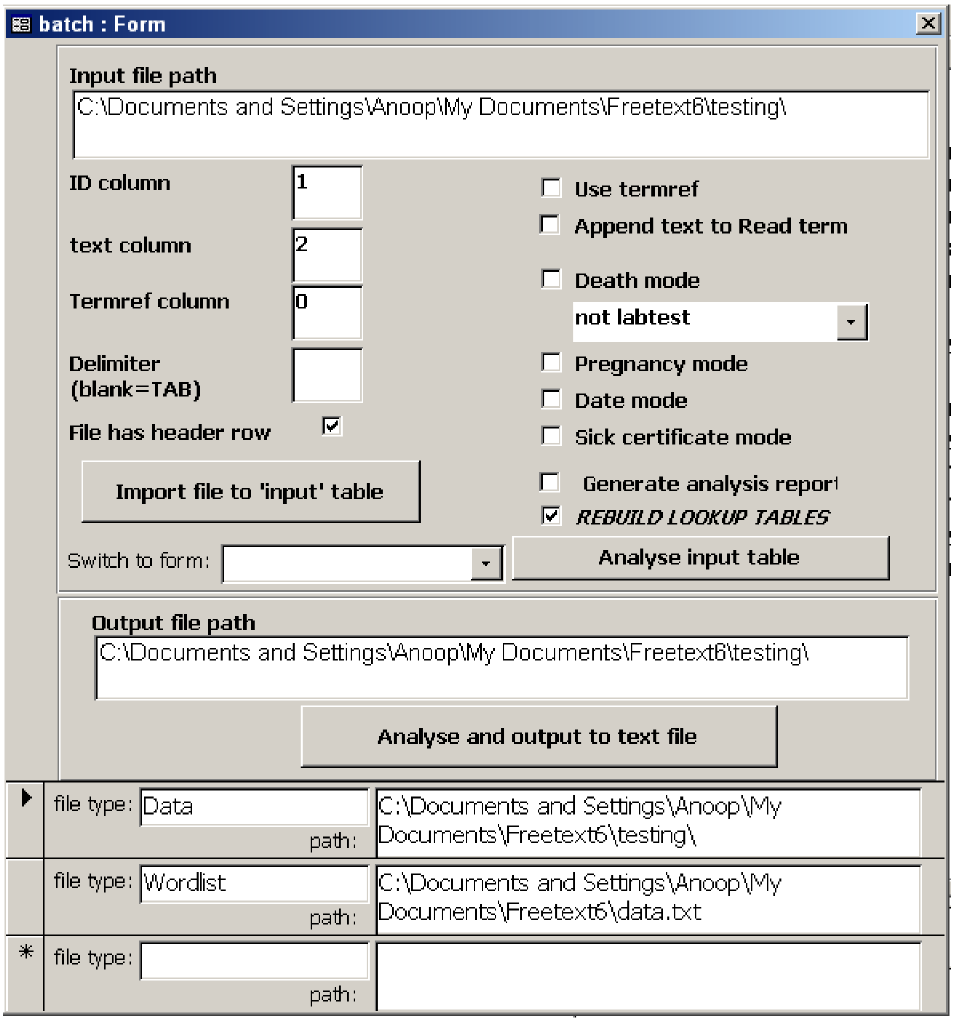

Figure 5: Form for selecting batch analysis options

3.5. Importing texts to the input table

`Text ID` and **`termref`** are optional. If the file does not contain either of these variables, set the relevant column to 0. Ids will be generated automatically if not provided. If IDs are provided, they must be unique. When a file is imported, existing data in the **`input`**, **`debug`** and **`output`** tables are erased.

3.6. Using text files

Type the full filepath of the input and output files in the boxes provided. The options to the right of the table apply when analysing the input table or when using text files. The output file format is tab delimited: **`id, order, data type, attribute, value`**. Note that a single input row can generate several output rows, which are numbered in the **`order`** field.

4. Examples of analysis

4.1. Normal mode

"re,pill on loestrin and antibiotics could she be pregnant ? will wait 1 week before starting ovran" →

1	READ	query	283679	PREGNANT
2	DURA_wks_		1	

"wound still sloughy at the bottom. cleaned and dressed w idoflex and dd, see 2/7" →

1	READ		305620	WOUND(S)
2	DURA_days	followup	2	

"160/90 SEE 1/12" →

1	LABS	sysbp	160
2	LABS	diabp	90
3	DURA_mths	followup	1

"warfarin 4mg od - inr 3.3 - rev 2/52" →

1	LABS	inr	3.3
2	DURA_wks_	followup	2

4.2. Append mode

The original Read or OXMIS term is in *italics*

ALLERGY "PENICILLIN RASH" →

1	READ	305707	ALLERGY PENICILLIN

Osteoarthritis "right knee. Advised weight reduction, gentle exercise etc. Paracetamol for pain relief." →

1	READ	244091	Knee osteoarthritis NOS
2	READ	309078	Pain

Chest pain "CHEST WALL" →

1	READ	309182	Chest wall pain

4.3. Death mode 'D'

"1a mi 1b coronary atherosclerosis 2 renal impairment, diabetes" →

1	READ	deathcause1a	298318	Acute myocardial infarction
2	READ	deathcause1b	298326	Coronary atherosclerosis
3	READ	deathcause2	234604	Renal impairment
4	READ	deathcause2	303256	DIABETES

"died on 25/7/04. cert at 18:30. R.I.P. Sudden unexpected death - Refer to coroner." →

1	READ		302004	DIED
2	DATE_full	deathdate	25-Jul-2004	
3	DATE_time		18:30	
4	READ		230556	Death
5	READ		296901	Referral to coroner

4.4. Lab test mode 'L'

Gamma - G.T. level "Original result: 'GGT' = 19(5 - 50)" →

1	LABS	19

Red blood cell distribution width "*** NUMERIC VALUE SUPPLIED: = 13.3 ***" →

1	LABS	13.3

4.5. Investigation result mode 'N'

Urinalysis - general "nad" →

1	LABS	nad

4.6. Pregnancy mode ‘P’

Antenatal care “28/40+4 no problems, fundus=dates, fmf, dipstick neg, BP 124/72” →

1	LABS	gest	28
2	LABS	sysbp	124
3	LABS	diabp	72

PREGNANT “34wks” →

1	LABS	gest	34

4.7. Sicknote mode ‘S’

MEDICAL CERTIFICATE “6 month” →

1	DURA_mths	sicknote	6

MED3 - doctor’s statement “3 weeks injured ankle” →

1	DURA_wks_	sicknote	3		
2	READ			218088	Other ankle injury

4.8. Time or date mode ‘T’

Date ceased smoking “23.8.97” →

1	DATE_full	date	23-Aug-1997

Patient date of birth “15 February 1945” →

1	DATE_full	date	15-Feb-1945

5. Attributes

5.1. Read terms

deathcause1a, deathcause1b, deathcause1c Cause of death – death certificate categories; e.g. ‘1a) MI b) coronary atheroma’ → ’MI’ has attribute `deathcause1a`; ‘coronary atheroma’ is `deathcause1b`

deathcause1c

deathcause1

deathcause2

deathcause Specifically stated as cause of death; e.g. "Cause of death: bronchopneumonia"

negative Associated clinical term is negative, e.g. "not cancer"

family Clinical term is associated with family member, not patient e.g. "wife has cancer"

negfamily negative family history; e.g. "no family history of stroke"

pmh past medical history e.g. "asthma age 7"

negpmh negative past medical history e.g. "no previous MI"

query Uncertainty about diagnosis ('query' or 'rule out'), e.g. "rule out MI"

dueto previous condition was caused by this condition, e.g. "MI due to atherosclerosis"

causing this condition was caused by previous condition e.g. "Septicaemia complicated by renal failure"

5.2. Dates

certdate or time when death was certified

admitdate admission or readmission date

dischdate discharge date

deathdate death date or time

lmp last menstrual period

dob date of birth (date of birth is not available in GPRD for confidentiality reasons. However it may be useful for verification exercises requiring record linkage, such as linking to death certificates or the mother-baby link.)

edd expected date of delivery

datenext date refers to next Read term (e.g. "1990 stroke")

dateprev date refers to preceding Read term (e.g. "MI in 1982")

followup follow up date

sicknote any date in a medical certificate entry (may be start or end date of certificate)

Only `certdate` and `deathdate` are allowed to be times. If the program is not operating in death mode, no attempt is made to extract times. This is to reduce the risk of a number (e.g. a test result) being incorrectly interpreted as a time.

5.3. Duration

duranext duration refers to next Read term

duraprev duration refers to preceding Read term

followup follow up time (e.g. "see in 3 months")

age e.g. "this 40-year-old man"

ageprev age at event relating to previous Read term, e.g. "diagnosed with asthma aged 10"

sicknote e.g. "*MED3* 1 week"

5.4. Lab tests

Note that laboratory results are not extracted in 'Death' analysis mode.

calcium

cholestesterol

cobalamin Vitamin B12

creatinine

diabp, sysbp Blood pressure. The program recognises a format such as '150/80' without 'blood pressure' stated explicitly, but only if the systolic pressure is higher than diastolic and both are in sensible ranges (`sysbp` 80–230, `diabp` 40–150)

esr Erythrocyte sedimentation rate

fbc Full blood count

ferritin

folate

gest Gestational age (duration in weeks, less than 43. In 'pregnant' mode, the program will interpret any duration in weeks as gestational age, as long as it does not have another attribute, and there no different duration in the text. Fractions are ignored by the function **`strfunc.get_date`** which converts durations into a structured format, e.g. "32/40 + 6" is converted to '32 weeks'.)

glyhb HbA1c

haemoglobin

hdl HDL cholesterol

inr International normalised ratio

ldl LDL cholesterol

mcv Mean cell volume

pefr Peak flow ('predicted' or 'best' peak flow is ignored)

platelets

pulse pulse rate in beats per minute, must be within the range 20 to 300

tetrathyroid Thyroxine, T4

trithyroid T3

tsh Thyroid stimulating hormone

triglycerides

urea

wbc White blood cells, leucocytes (may apply to blood, urine or other fluids, depending on associated Read term)

The function `pd.correct_attr` controls whether an attribute is correct for a particular meaning, except for `LABS` data type where this check is not used. It is possible to introduce a new `LABS` attribute without modifying the program code.

6. How to modify the algorithm tables

6.1. Form terms2: terms

The table of Read Terms is used for three purposes:

1. To decide the analysis options for the current text, based on its associated termref
2. To enable the free text to be appended to the text of the Read Term, in order to produce a complete statement which is similar to what the doctor would have seen when using the practice management system. This applies when using the analysis option 'Append'.
3. To derive the list of terms to which text can be converted (those with Include=TRUE).

The form terms2 displays the terms table and allows manipulation of the synonym table. By default it displays the entire table but it can be filtered on term, `termref` or `std_term` (standardised term).

6.2. Form terms2: synonyms

The table of synonyms is used for three purposes:

1. Searching for candidate text in s1 to see if it is possibly part of a clinical term (from sub `freetext_core.initial_search`).
2. Generating a variation of the free text by substituting s1 components by their s2 links (function `list.expand`).

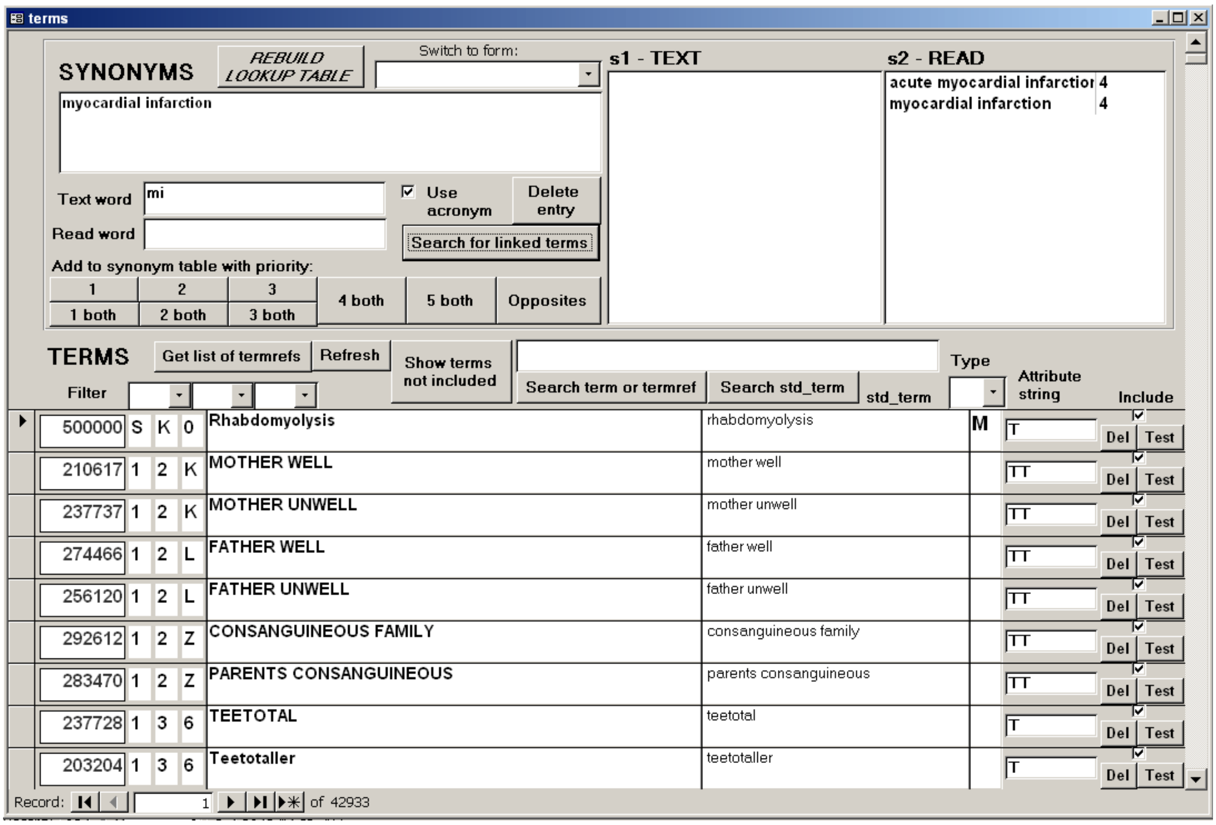

Figure 6: Form terms2

3. Trying to match a Read term segment to part of the free text, as part of a test of match accuracy (function **`freetext_core.readscore`**). This comparison is between the original free text segment (including ignorable words) and the Read term.

6.2.1. Data entry fields

Text word word or phrase to search for in unstructured text

Read word word or phrase to search for in Read term

Use acronym terms such as 'mi – myocardial infarction' will also be added as 'm i – myocardial infarction'.

6.2.2. Buttons

Delete entry deletes the link between Text word and Read word

Search for linked terms searches synonym table for s2-READ links to the entry in Text word, and also s1-TEXT links to the entry in Read word, and displays both sets of results. In the results the s1-TEXT terms are not directly linked to the s2-READ terms, but the s1-TEXT terms are linked to the Read word and s2-READ to Text word.

1, **2**, **3** adds the match between Text word and Read word with the selected priority

Opposites adds the match with a priority of -100

1 both, **2 both** **etc.** adds the match and also the same match with Read word and Text word swapped around.

Rebuild lookup tables regenerates the temporary text file used for rapid loading of the lookup tables. This needs to be done before analysing text, if any changes have been made to the tables..

6.2.3. How to add a new synonym

- Type the text phrase in the Text word box (lower case, with one space between words, no punctuation).
- Type the standardised Read phrase to which it will match in the Read word box.
- Click one of the buttons (e.g. 4, 5 etc.) under 'Add to synonym table with priority' to add the link to the table. If the link already exists, it will be over-written with the new priority.
- Just before analysing the next text, click Rebuild lookup tables to ensure that all the changes are implemented.

Priorities

5 exact match e.g. 'chronic obstructive pulmonary disease' $\leftrightarrow$ 'copd'; the match is automatically made both ways.

4 almost exact match e.g. 'abnorm' $\leftrightarrow$ 'abnormal'; the match is automatically made both ways.

3 moderate match (e.g. text phrase is mis-spelt or is more specific than Read phrase); e.g. 'b pne' $\rightarrow$ 'bronchopneumonia'; 'carcinoma' $\rightarrow$ 'malignant neoplasm'

2 non-standard abbreviation or distorted form; possible one-way match (Read phrase is wider than text phrase); e.g. 'rsi' $\rightarrow$ 'repetitive strain injury'

1 loosely associated (Read phrase much wider than text phrase); e.g. 'foot' $\rightarrow$ 'lower limb'

-100 Opposite; e.g. 'left' $\neq$ 'right', 'finger' $\neq$ 'toe'

When searching for the best Read term match, links with higher priority are used where possible.

When the function `list.expand` searches on s1 for alternative words, the search text has been stripped of 'ignorable' words, so for example if the free text contained 'A and E', the search text would be 'a e'. This means that for any synonym link involving 'a and e' there should be a corresponding link with 'a e'. This additional match is made automatically, with priority 1, whenever a synonym link is added 'both ways'.

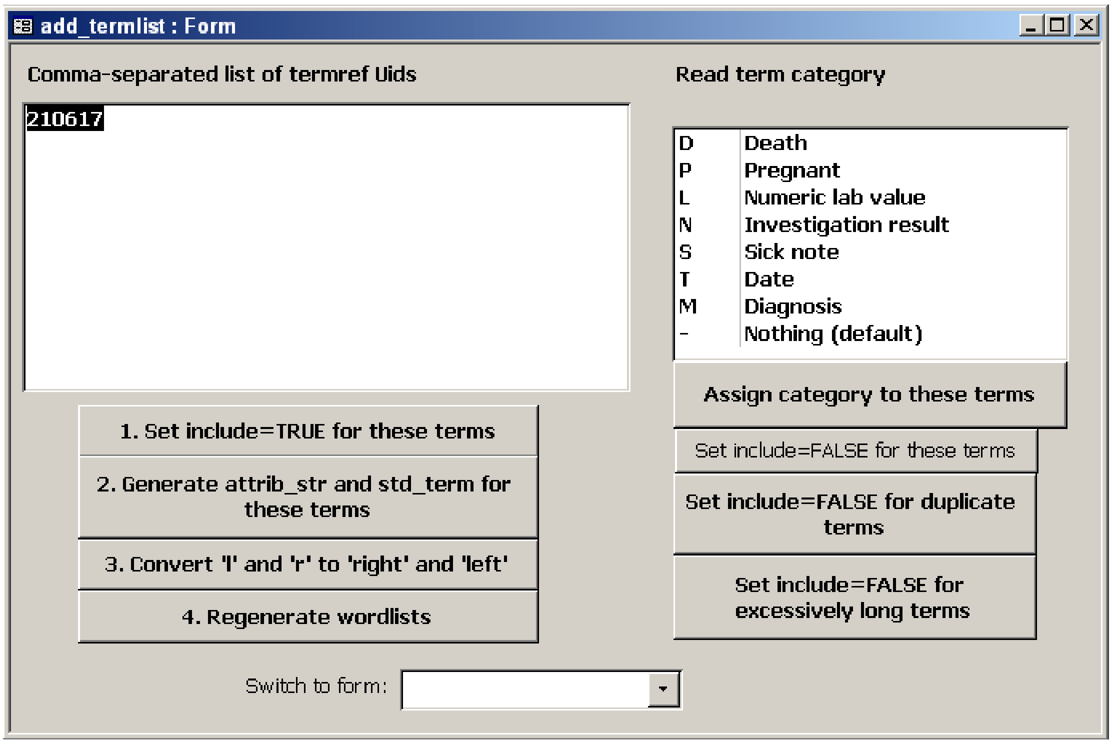

Figure 7: Form add_termlist

6.3. Form add_termlist

Used for setting the read term type, and adding terms for conversion (see Figure 7).

6.3.1. Set Include=TRUE for a single term

1. In the terms2 form, tick the Include check box for each term to add.
2. Open add_termlist and click 4. Regenerate wordlists.

After setting Include=FALSE using the Delete button on the terms2 form, there is no need to regenerate the wordlists.

6.3.2. Set Include=TRUE for a set of terms

1. Either paste a list of the termref Uids (comma separated) in the text box, or use the `Terms2` form and filter it to show only the required terms, then click Get list of termrefs.
2. Click Set include=TRUE for these terms.
3. If these terms have blank std_term or attribute strings, click 2. Generate attrib_str and std_term for these terms then 3. Convert 'l' and 'r' to 'right' and 'left'.

4. Click 4. Regenerate wordlists. This regenerates the **singlewords** and **doublewords** tables using the **std_term** in the

5. 'Compact and repair' the database using the main Microsoft Access menu. This should be carried out regularly, and especially after modifying a large amount of data.

6. After setting Include=TRUE for a large number of terms, run the following procedures:

   a) Set Include=FALSE for duplicate terms

   b) Set Include=FALSE for excessively long terms.

6.3.3. Set the Read term category for a set of terms

1. Generate the set of termlist Uids as in step 1 above.
2. Click on the Read term category to set. 'Nothing' sets the category to blank.
3. Click Assign category to these terms.

6.3.4. To regenerate the terms table

Once **std_term** and **attrib_str** have been generated for all the terms, there would be no reason to change them unless an error is discovered. However if it is necessary, it can be done by deleting any termrefs in the box, then clicking buttons 2, 3, and 4 in order. Generating the **attrib_str** and **std_term** for the entire table takes about 30 minutes.

6.4. Form attrib2

The form **attrib2** displays the contents of the **attrib** table, which contains a list of word patterns which are used to derive the context of Read terms, lab results and dates (see Fig. fig:attrib2form). The boxes and buttons at the top are used to enter new terms. Existing terms can be edited directly in the main body of the form. The Filter list button can be used to restrict the patterns displayed to particular attributes or search positions for ease of use.

The attribute search takes place *after* detection of dates and durations but *before* analysis of Read terms. However, words which might form part of a Read term are marked with data type **CLIN**. Lab results are extracted during the attribute search. Some attributes are further manipulated by the core program.

Each row of the **attrib2** table contains up to 5 words with punctuation, and the attributes to which they map.

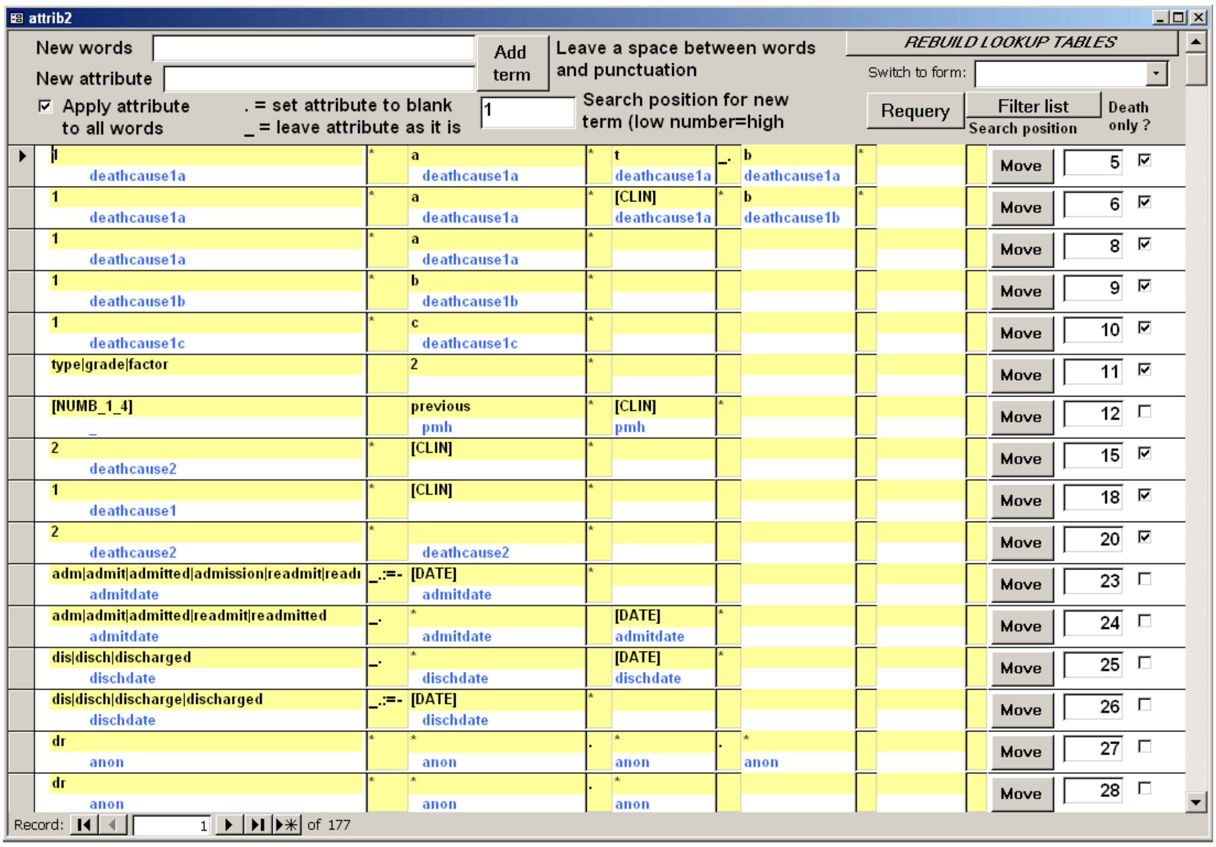

Figure 8: Form for editing the attrib2 table

6.4.1. Buttons

Add term adds a new pattern with words and attributes as in the appropriate boxes (see below for format)

Requery refreshes the query for the form

Filter list brings up a dialog box asking which attribute to filter by. Type in the attribute and click OK (* can be used as a wildcard; e.g. 'death*' would search for 'deathcause1a', 'deathdate' etc.).

Move brings up a dialog box asking for the new search position of the pattern. Type the new position or 999 to delete the pattern. On clicking OK, the patterns are re-arranged automatically.

Rebuild lookup tables regenerates the temporary text file used for rapid loading of the lookup tables. This needs to be done before analysing text, if any changes have been made to the tables.

6.4.2. Format of attribute patterns

The pattern can match up to 5 words. For each word you can specify a choice of words or data types which are acceptable for the match. If a data type rather than a specific word is given, it must be in square brackets (see Table 4).

Example or code	*Meaning*
`*`	Any word or punctuation
`thisword`	Specific word: 'thisword'
`this|that|[NUMB]`	Either 'this', 'that' or a number
`2`	Specific number: 2
`[CLIN]`	Any word which might be part of a Read term
`[IGNO]`	An ignorable word, e.g. 'and', 'as', 'at', 'by'
`[NUMB]`	Any word with data type 'number', which includes some lab results such as 'normal'
`[NUMB_70_230]`	Number between 70 and 230
`[DATE]`	Date in any format
`[DATE_full]`	Full date
`[DATE_year]`	Year
`[DURA]`	Duration in any format
`[DURA_wks_]`	Durations in weeks
`[ATTR attribute]`	Any word with specified attribute

Table 4: Attribute patterns

Punctuation is shown in the small boxes to the immediate right of the word. The asterisk (*) is a wildcard character which meany that any punctuation, or blank is acceptable. Underscore (_) represents no punctuation. For other punctuation elements, if the punctuation is part of the pattern, it is accepted. For example, if the pattern is `_:=` then ':', '=', ':=' or no punctuation would be allowed.

Attributes can be assigned to each of the words in the pattern. The following special codes can also be used:

anon Anonymise; do not analyse this part of the text (e.g. if it may contain the name of a doctor, patient or hospital)

ignore Ignore

normalrange Ignore any following lab values unless they have their own attribute

possiblity Ignore any following diagnoses unless they have their own attribute

_ (underscore) Retain current attribute

. (full stop) Set attribute to blank

DATATYPE attribute Set attribute to **`attribute`** and data type to **`DATATYPE`** (e.g. **`LABS inr`**; **`DURA_wks_ followup`**)

The *search position* is the order in which the patterns are used; those with higher search positions are used first. Attributes of patterns which are used later (i.e. with lower numbers) can overwrite attributes set by earlier patterns.

The death only check box means that this pattern is only used if the text is being analysed in 'death' mode.

6.4.3. Data entry fields

To add an attribute: type the text in the top text box, and the attributes below it. Leave a space between each word and punctuation.

New words type in the words for the new pattern, leaving one space between each word and the next, or a word and punctuation (e.g. tsh * [NUMB])

Apply attribute to all words (check box) tick if all the words in the pattern have the same attribute, and type the attribute once only in the 'new attribute' box.

New attribute either word by word or just one for all words. Attributes can only be entered as one word using this system. If an entry is to have two words (e.g. 'LABS tsh'), where the first word is the data type and the second is the attribute, it has to be altered manually in the form after first entering it using the automatic system.

Search position Search position for new term. If this position is already taken, the existing patterns are moved out of the way to make room.

6.4.4. How to add a new pattern for an existing attribute

- Type in the pattern in the 'New words' box. Leave a space between words and punctuation. Use the codes described above.
- Type in the new attribute, with one space between words. If the new pattern has the same attribute for all words, tick the check box apply attribute to all words.
- If the rule should set the data type as well as the attribute, modify the entry in the attribute table afterwards.

For example, "chol `[NUMB]`" → `LABS cholesterol`:

1. Add attribute: words = 'chol * [NUMB]'; new attribute = `cholesterol`, Apply attribute to all words = TRUE; Search position = whatever desired
2. Scroll down the form (or use filter list) to find the new pattern, and change the second attribute entry from `cholesterol` to `LABS cholesterol`. This entry corresponds to `[NUMB]`, the actual value of the lab result.

6.4.5. How to add a new attribute

- Add the attribute pattern using the attrib2 form as described above.
- For data type LABS this is all that is required. For other data types, modify the function correct_attr in module pd to allow the new attribute for that particular data type.
- Modify the program code if it is necessary to set the attributes in a way which is not possible using the `attrib` table.

6.5. Other tables

The other core tables have simple data entry forms corresponding to their names; please see the appropriate paragraph in subsection 2.4.

checkterms see subsubsection 2.4.4 on page 15.

ignore, ignore_phrase see subsubsection 2.4.5 on page 17.

Read_attr1, Read_attr2 see subsubsection 2.7.2 on page 21.

7. Testing the algorithm

7.1. Using the freetext form

The `freetext` form can be used to analyse a single text or check the results of a set of texts in turn. For a general guide to using this form, see subsection 3.1 on page 22. This section describes the data fields for recording conversion accuracy.

Each output data element has the following check boxes, which correspond to fields in the output table (see Table 3 on page 20):

Happened: auto For READ terms, this is whether the program thinks the event actually happened to this patient, as evidenced by the attribute. It is negative if the attribute is 'negative', 'family', 'query', 'negfamily', 'negpmh'. This variable is set by sub `results_output` (see subsection 18.6 on page 84) and can not be altered manually from the form. For dates, this is TRUE if the date has an attribute. For Lab results it is always TRUE.

Happened: actual This is initially set to be the same as 'Happened: auto' but can be altered manually. If it does not correspond to 'Happened: auto' it means the computer has made an error.

Important Whether this output entry should be ignored because it is a duplicate. This is set to TRUE as default.

Correct attribute For READ terms, whether the attribute is the best possible choice. Default=TRUE. (If the attribute is completely wrong it might also affect 'Happened: actual'.) Dates and Lab results are considered to be wrong if the attribute is wrong.

Correct value For READ terms, whether the chosen Read term is the most specific and accurate term available. Default=TRUE. If the choice is completely wrong, 'Happened: actual' should also be set to FALSE.

There are also several fields in the input table (see also Table 2 on page 20):

Comments Click the button to add a comment.

Read missed, Dates missed, Labs missed Number of pieces of data not detected in each category. Click the buttons to increase the number by 1.

Original term false If the original term did not apply to this patient as evidenced by the free text (e.g. Read term 'DEATH' with free text 'of mother').

New term better Whether the new term combining the original Read term and free text is more specific than the original Read term (see subsection 4.2 on page 25 for examples).

7.2. Analysis reports

7.2.1. Overall analysis

Module freetext_core contains a global variable 'debug_string', which can be used to store an analysis report if this option is chosen. When analysing a single text using the freetext form interface, the program automatically uses the debug option, and when analysing a batch of texts from the input table or a text file, this option is switched off to save time.

Various functions add entries to debug_string to document the stages of analysis. Line breaks are inserted by appending ASCII character 13 then character 10. This example is based on the following free text:

"another hospital admission- still having daily symptoms and using salbutamol ++++.To increase symbicort to 200/6 2pufbd and rev 1m if no improvement.Man /plan discussed."

The analysis report contains the following items (only selected portions of the analysis report are shown, to save space):

1. Heading `INITIAL_SEARCH, ATTRIB.PD_SEARCH2`
2. Attribute patterns which match to the text (show attrib phrase 'Matches to:' text phrase). Example:

   ```
   Attrib phrase (search position 424): 1|[ATTR followup]
   2|[DURA]|[DATE]*
   Matches to: rev 1
   ```

   Utility: shows which attribute patterns were used and the text they recognised. If a context is detected incorrectly, this part of the report shows whether the context was detected in

the first place. Modification or addition of patterns to the attribute table might reduce errors seen at this stage.

3. Listing of arrays in module pd, containing words, punctuation, attribute and meaning in separate arrays. This listing is after the words have been given provisional data types, and the attributes have been allocated according to the arrays. Example:

```
Word : Punctuation : Meaning : Attribute
another :  : CLIN another :
hospital :  : CLIN 3 2 :
admission : - : CLIN 3 2 :
still :  : IGNO : ignore
having :  : WORD having :
daily :  : WORD daily :
symptoms :  : CLIN 2 1 :
```

Utility: 'Word' column shows text after remove_ignore_phrases and initial_process. 'Meaning' shows initial allocation of data types, particularly dates (function strfunc.get_date is called to try to extract a date from every sequence of up to 5 words). 'Attribute' shows the result of attrib.pd_search2 i.e. after recognition of patterns using the attribute table.

4. Heading `ATTRIB_SEARCH, ANALYSE_PD`

5. Show each sequence of words of data type 'CLIN' (possibly with 'IGNO' or 'NUMB' words in between) tested i.e. words which might be part of a Read term. Each section headed 'List of candidate terms' is the record of a single call to the function 'bestmatch' (see subsection 12.3 on page 54). This function is called with a sequence of up to 8 contiguous words from the text, and returns the match with the highest readscore or the first match found with readscore higher than threshold_high. If a Read match is found, the report contains the readscore, termref Uid, Read term text, and the text to which it matches. Example of listing:

```
List of candidate terms
0 0  [another hospital]
0 0  [another hosp ]
0 0  [another hospital care ]
0 0  [another hosp l ]
Total 3 terms

List of candidate terms
0 0  [another]
Total 0 terms

List of candidate terms
0 0  [hospital admission]
100 309362 HOSPITAL ADMISSION
          [hospital admission]
```

```
Total 1 terms
```

Utility: Shows which sequences of words were chosen for conversion to Read terms (sub freetext_core.analyse_pd). Shows which alternative texts were generated by using the synonym table. Shows the converted text from which the Read term match was made, and the readscore (calculated with reference to the original text). Errors at this stage might be reduced by adding or editing synonym entries.

6. Listing of pd arrays, now containing linked Read termref Uids alongside text. Example:

```
Word : Punctuation : Meaning : Attribute
another :  : CLIN another :
hospital :  : READ 309362 100 :
admission : - : READ 309362 100 :
still :  : READ 309362 100 :
having :  : WORD having :
⋮
and :  : IGNO : ignore
rev :  : WORD rev : followup
1 :  : DURA_mths 1 : followup
m :  :  : followup
if :  : WORD if : followup
no :  : IGNO : possibility
```

Utility: shows the original text which was linked to a Read term.

7. Heading: `PD.COMPRESS, PD.CHECK_COMPRESSED`

8. Listing of pd arrays, now containing one row per structured data element. Ignore the ’Word’ and ’Punctuation’ columns - at this stage the original text entry is no longer used by the program. Example:

```
Word : Punctuation : Meaning : Attribute
another :  : READ 309362 100 :
hospital :  : DURA_mths 1 : followup
```

Utility: useful for checking the effect of the sub pd.check_compressed (see subsection 13.2 on page 57), which rejects attributes and/or the data elements themselves if they do not make sense.

7.2.2. Readscore

The form **`terms2`** contains a button next to every term which can be used to test the readscore function (see subsection 9.10 on page 49). This calls up a dialog box for entry of the free text segment to test the Read term against. The procedure involves initialising the arrays as for a normal analysis. However, rather than trying out possible Read term matches, only the term of interest is used, and the score is calculated.

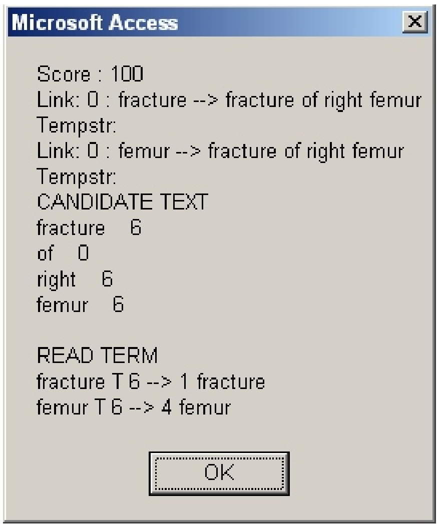

Figure 9: Dialog box displaying results of readscore test

The score is based mostly on the proportion of non-ignorable words in the candidate text mapped to a word in the Read term and vice versa, with a few extra points available for the priority of synonym matches, and whether ignorable words were mapped also. The result is displayed in a dialog box, showing the link made to each word of the original text and the Read term.

Example: testing 'fracture of right femur' with OXMIS term 'FRACTURE FEMUR'. The standardised Read term is 'fracture femur'. The word 'right' in the text is ignored as long as there is no word 'left' in the Read term.

The readscore is interpreted in view of the following thresholds, which are declared as constants in the module list: **`threshold_high`** = 91; if match with this readscore is found, the program does not search for other matches. **`threshold`** = 87; minimum readscore for a satisfactory match

7.3. Aggregate reports

The **`reports`** form provides a convenient way to organise a set of queries and calculations to give an overall report of the algorithm performance. The data is stored in the table **`reports`**, which has the following fields:

ID autonumber

sqltext Either:

- An SQL 'SELECT COUNT' query which returns the answer in a variable called **`result`**
- A calculation using the results of other queries. The formula must start with '=', and references to previous results must be enclosed in braces { }.

result Output from the sqltext query or calculation

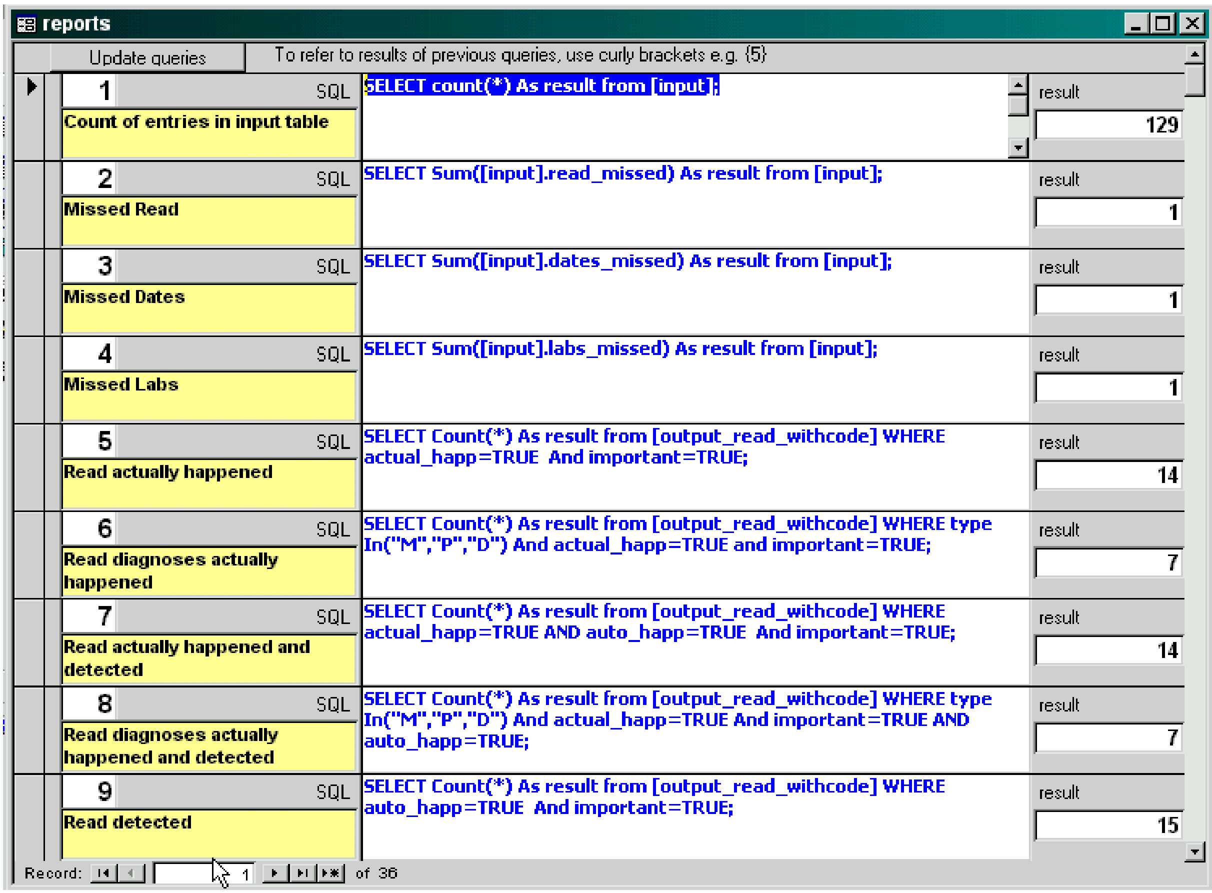

Figure 10: Reports form

description Description of the outcome variable

The ‘reports’ report displays the ID, description and results from the `reports` table, in a format suitable for printing. When the report is opened, it prompts for analysis options and a description of the test sample, which are then displayed in the report.

8. Program development

8.1. Background

A computer program (`freetext3.mdb`) was developed to extract structured information from free text entries associated with death entries. The output was in the form of Read Clinical Terms with an associated context flag denoting the death certificate category of the diagnosis, or whether it was due to another condition, whether it was uncertain (‘query’) or negative or referred to someone other than the patient (‘family’). Dates and times were also extracted. The algorithm was tested on a random sample of texts containing 625 diagnoses, and it converted 86% to the correct Read Term and context flag.

8.2. Further development

The program was further developed in July-November 2005 for use with other types of free text and extract information on referrals and hospitalisations. This documentation refers to the most updated version of the program: freetext10.mdb.

8.3. Major changes

8.3.1. Information extracted

Symptoms, examination findings, tobacco and alcohol consumption, immunisations, hospitalisation and referrals. (Therapeutic procedures and contraception are currently not converted to Read terms).

8.3.2. User interface

Forms re-designed for ease of use, and to make it easy to view the intermediate results of analysis, and add attribute patterns and synonyms to increase accuracy in the future.

8.3.3. Changes made to increase speed

1. All tables are now loaded into RAM before analysis. Lists are searched using direct reference to sorted lists held in arrays, rather than using SQL to access the database tables. A further change made in Jan 2006 was to export all the lookup tables to a text file, which is quicker to load and makes it quicker to analyse a single text. It will also make it possible to produce an 'end-user' version of the program which does not use database tables at all. The text file has to be regenerated after making any changes to the tables.
2. A new table 'doublewords' was added, which consists of all pairs of two words which appear in Read terms, and their associated termref Uids. Previously the program would generate a set of termref Uids from each individual word, and then calculate the intersection of the two sets in order to generate the list of termref Uids present in both words.
3. The old wordlist table was renamed 'singlewords'. A new 'wordlist' table was generated which contained just a list of all the words in the Read terms, and was used for initial spelling correction.
4. A modified function enabling single character spelling mistakes in long words to be corrected.

Part II. GUIDE TO VISUAL BASIC CODE

The source code and lookup tables are currently available on request from the authors but we aim to make them available on the internet. The program is licensed under under the GNU General Public License Version 3. The Visual Basic documentation was produced using the 'Documentation' Visual Basic program (included with the source code for this project) which extracts comments, arguments and function calls from Visual Basic code and produces LaTeXdocumentation.

9. Module freetext_core

Main subs and functions for the program

9.1. Global variables and constants

Const `wordmatchthreshold` = 0.73 (*used by readscore*)
`debug_string` – String (*stores analysis report for an individual text, when running in debug mode*)
`death` – Boolean (*whether Read term implies death*)
`gest` – Boolean (*whether Read term refers to weeks gestation*)
`spell` – Boolean (*whether to use spelling correction*)

9.2. Sub init_all

Initialises all the arrays from the data text file. If filepath is not provided, it is obtained from the table 'file_location'. Arrays must be loaded in the dame array that they are saved in the file.

Arguments: `filepath` – String (Optional)

Subs and functions called: `attrib.infile` subsection 10.3 on page 50
`synonym.infile` subsection 15.2 on page 71
`wordlist.infile` subsection 17.2 on page 77
`terms.infile` subsection 16.2 on page 75
`checkterms.infile` subsection 11.3 on page 52
`freetext_core.outfile_all` subsection 9.3 on page 46

Called by: `in_out.filepath` subsection 18.1 on page 83
`in_out.do_input_table` subsection 18.5 on page 84
`in_out.do_text_file` subsection 18.7 on page 85

9.3. Function outfile_all As Boolean

Initialise all arrays from database tables, and (if dont_export is False or not given) save them to a text file. The text file is specified in the table file_location, with the [file type] of 'Wordlist'. The value returned is whether or not the new file was successfully generated

Arguments: `filepath` – String (Optional)
`dont_export` – Boolean (Optional)

Subs and functions called: `attrib.init` subsection 10.2 on page 50
`synonym.init` subsection 15.4 on page 71
`wordlist.init` subsection 17.4 on page 78
`terms.init` subsection 16.4 on page 75
`checkterms.init` subsection 11.2 on page 52
`attrib.outfile` subsection 10.4 on page 51
`synonym.outfile` subsection 15.3 on page 71
`wordlist.outfile` subsection 17.3 on page 77
`terms.outfile` subsection 16.3 on page 75
`checkterms.outfile` subsection 11.4 on page 52

Called by: `freetext_core.init_all` subsection 9.2 on page 45
`maintenance.make_wordlist` subsection 19.10 on page 88

9.4. Sub main_termref

if append = True, text is appended to Read term (to appear as it would on the doctor's computer)

Arguments: `instring` – String
`Termref` – Long
`spell_` – Boolean (Optional)
`debug_` – Boolean (Optional)
`append_term` – Boolean (Optional) (ByVal)

Subs and functions called: `terms.read_type` subsection 16.7 on page 76
`terms.std_term` subsection 16.8 on page 76
`strfunc.in_set` subsection 14.5 on page 66
`freetext_core.main` subsection 9.5 on page 47
`pd.mean` subsection 13.10 on page 59
`strfunc.dissect2` subsection 14.13 on page 69
`pd.Attr` subsection 13.9 on page 59
`pd.remove` subsection 13.24 on page 63

Called by: `in_out.filepath` subsection 18.1 on page 83
`in_out.do_input_table` subsection 18.5 on page 84
`in_out.do_text_file` subsection 18.7 on page 85

9.5. Sub main

Main analysis, using all the options

Arguments: `instring` – String (ByVal)
`death_` – Boolean (Optional)
`pregnant_` – Boolean (Optional)
`debug_` – Boolean (Optional)
`labtest` – String (Optional)
`spell_` – Boolean (Optional)
`date_only` – Boolean (Optional)
`termstring` – String (Optional)
`append_term` – Boolean (Optional)
`sicknote` – Boolean (Optional)

Subs and functions called: `freetext_core.readscore` subsection 9.10 on page 49
`wordlist.remove_ignore_phrases` subsection 17.23 on page 82
`pd.init_read` subsection 13.21 on page 63
`freetext_core.initial_search` subsection 9.6 on page 47
`attrib.pd_search2` subsection 10.6 on page 51
`pd.show_all_2` subsection 13.7 on page 58
`freetext_core.attrib_search` subsection 9.7 on page 48
`freetext_core.analyse_pd` subsection 9.8 on page 48
`pd.compress` subsection 13.4 on page 58
`pd.check_compressed` subsection 13.2 on page 57
`checkterms.check_all` subsection 11.5 on page 52

Called by: `freetext_core.main_termref` subsection 9.4 on page 46
`in_out.filepath` subsection 18.1 on page 83
`in_out.do_input_table` subsection 18.5 on page 84
`in_out.do_text_file` subsection 18.7 on page 85

9.6. Sub initial_search

tested 3 Jan 03; analyses pd for Read terms, synonyms, attributes and dates

Arguments: `debug_` – Boolean (Optional)

Subs and functions called: `pd.max` subsection 13.28 on page 64
`pd.part_nopunc` subsection 13.16 on page 61
`strfunc.get_date` subsection 14.2 on page 65
`pd.part_punc_nospace` subsection 13.17 on page 61
`pd.add_mean` subsection 13.15 on page 61
`synonym.get_search_summary` subsection 15.8 on page 72
`pd.text` subsection 13.25 on page 64
`wordlist.ignorable` subsection 17.20 on page 82

pd.add_attr subsection 13.14 on page 60
strfunc.is_numeric subsection 14.14 on page 70
wordlist.wordsearch subsection 17.19 on page 81
pd.set_text subsection 13.26 on page 64

Called by: **freetext_core.main** subsection 9.5 on page 47

9.7. Sub attrib_search

now assign attributes to actual read terms, dates etc.

Arguments: **debug_** – Boolean (Optional)

Subs and functions called: **pd.max** subsection 13.28 on page 64
pd.Attr subsection 13.9 on page 59
strfunc.in_set subsection 14.5 on page 66
pd.mean subsection 13.10 on page 59
pd.text subsection 13.25 on page 64
pd.punct subsection 13.27 on page 64
pd.set_attr subsection 13.12 on page 60

Called by: **freetext_core.main** subsection 9.5 on page 47

9.8. Sub analyse_pd

analyses pd after the initial search; attempts to convert terms into readcodes.

Arguments: **debug_** – Boolean (Optional)
labtest – String (Optional)

Subs and functions called: **strfunc.in_set** subsection 14.5 on page 66
pd.mean subsection 13.10 on page 59
pd.Attr subsection 13.9 on page 59
pd.max subsection 13.28 on page 64
pd.punct subsection 13.27 on page 64
pd.text subsection 13.25 on page 64
list.bestmatch subsection 12.3 on page 54
pd.set_mean subsection 13.13 on page 60
strfunc.words subsection 14.4 on page 66
pd.set_attr subsection 13.12 on page 60

Called by: **freetext_core.main** subsection 9.5 on page 47

9.9. Function remove_ignorable As String

modified sep 04 and Oct 05. Removes ignorable words from a phrase. Requires one space between words; no punctuation

Arguments: `instring` – String (ByVal)
`remove_right_left` – Boolean (Optional)

Subs and functions called: `strfunc.numwords` subsection 14.7 on page 67
`strfunc.dissect2` subsection 14.13 on page 69
`wordlist.ignorable` subsection 17.20 on page 82
`strfunc.in_set` subsection 14.5 on page 66

Called by: `list.getlist` subsection 12.8 on page 56
`synonym.add` subsection 15.6 on page 72

9.10. Function readscore As Single

produces a score based on the accuracy and completeness of match between free text (from partdata) and candidate Read term. Score between 0 and 100

Arguments: `pd_start` – Long
`pd_fin` – Long
`Termref` – Long
`debug_` – Boolean (Optional)
`clear_memory` – Boolean (Optional)

Subs and functions called: `terms.std_term` subsection 16.8 on page 76
`strfunc.numwords` subsection 14.7 on page 67
`pd.part_nopunc` subsection 13.16 on page 61
`terms.attrib_str` subsection 16.9 on page 76
`pd.Attr` subsection 13.9 on page 59
`strfunc.dissect2` subsection 14.13 on page 69
`synonym.trylink_2` subsection 15.9 on page 73
`strfunc.words` subsection 14.4 on page 66
`pd.text` subsection 13.25 on page 64
`pd.true_` subsection 13.8 on page 59
`strfunc.in_set` subsection 14.5 on page 66
`wordlist.ignorable` subsection 17.20 on page 82

Called by: `freetext_core.main` subsection 9.5 on page 47
`list.getlist` subsection 12.8 on page 56

9.11. Function fuzzylink As Long

Whether the two words are almost the same (maximum one character difference). Assume the first character is the same and they differ in length by at most 1. Gives a score (letter position of difference, zero if too different

Arguments: `ref_word` – String
`test_word` – String

Subs and functions called: none

Called by: `wordlist.wordsearch` subsection 17.19 on page 81

10. Module attrib

10.1. Global variables and constants

Const `maxattrib` = 400
`w(5, maxattrib)` – String
`p(5, maxattrib)` – String
`a(5, maxattrib)` – String
`death_only(maxattrib)` – Boolean
`numwd(maxattrib)` – Long
`order(maxattrib)` – Long (*for debug purposes only*)
`num` – Integer

10.2. Sub init

initialises arrays for attribute search

Arguments: none

Subs and functions called: `attrib.dissect2_options` subsection 10.5 on page 51

Called by: `freetext_core.outfile_all` subsection 9.3 on page 46

10.3. Sub infile

Inputs everything from filenumber 1

Arguments: none

Subs and functions called: none

Called by: `freetext_core.init_all` subsection 9.2 on page 45

10.4. Sub outfile

Outputs arrays to filenumber 1

Arguments: none

Subs and functions called: none

Called by: `freetext_core.outfile_all` subsection 9.3 on page 46

10.5. Function dissect2_options As String

Counts the options and puts it at the front for future use by the dissect2 function when using these words: i.e. 3|word|another|option. Modified 25-oct-05 to do this for ALL words, not only those with options (e.g. if ony option it will be 1|thing).

Arguments: `instring` – String

Subs and functions called: none

Called by: `attrib.init` subsection 10.2 on page 50

10.6. Sub pd_search2

Death=true means include death certificate terms. New search strategy - by attrib term rather than by word in original text. Searches pd using attrib2 table; results are added to attribute fields of pd

Arguments: `debug_` – Boolean (Optional)
`death` – Boolean (Optional)

Subs and functions called: `pd.max` subsection 13.28 on page 64
`pd.matchpattern` subsection 13.18 on page 62
`pd.set_attr` subsection 13.12 on page 60
`pd.set_mean` subsection 13.13 on page 60
`strfunc.dissect2` subsection 14.13 on page 69
`pd.text` subsection 13.25 on page 64
`pd.part_punc_nospace` subsection 13.17 on page 61

Called by: `freetext_core.main` subsection 9.5 on page 47

11. Module checkterms

Added October 2005. Checks for occurence (or not) of words in the text to validate or invalidate some termrefs

11.1. Global variables and constants

Const **maxcheckterms** = 100
Termref(maxcheckterms) – String
Qualify(maxcheckterms) – String
Dequalify(maxcheckterms) – String
used – Long (*number of entries*)

11.2. Sub init

Loads entries from checkterms table into arrays

Arguments: none

Subs and functions called: none

Called by: **freetext_core.outfile_all** subsection 9.3 on page 46

11.3. Sub infile

Inputs everything from filenumber 1

Arguments: none

Subs and functions called: none

Called by: **freetext_core.init_all** subsection 9.2 on page 45

11.4. Sub outfile

Outputs arrays to filenumber 1

Arguments: none

Subs and functions called: none

Called by: **freetext_core.outfile_all** subsection 9.3 on page 46

11.5. Sub check_all

Carries out the actual checking

Arguments: **checkstring** – String
debug_ – Boolean (Optional)
sicknote – Boolean (Optional)
death – Boolean (Optional)
date_only – Boolean (Optional)

Subs and functions called: `pd.max` subsection 13.28 on page 64
`pd.mean` subsection 13.10 on page 59
`pd.set_attr` subsection 13.12 on page 60
`pd.Attr` subsection 13.9 on page 59
`pd.remove` subsection 13.24 on page 63
`strfunc.dissect2` subsection 14.13 on page 69
`checkterms.in_list` subsection 11.6 on page 53
`checkterms.if_qualify` subsection 11.7 on page 53
`checkterms.if_dequalify` subsection 11.8 on page 53

Called by: `freetext_core.main` subsection 9.5 on page 47

11.6. Function in_list As Long

returns the position of the termref in the table

Arguments: `in_termref` – Long

Subs and functions called: none

Called by: `checkterms.check_all` subsection 11.5 on page 52
`list.expand` subsection 12.4 on page 54
`list.display` subsection 12.6 on page 55

11.7. Function if_qualify As Boolean

whether one of the qualifying terms is present in the text

Arguments: `pos` – Long
`checkstring` – String

Subs and functions called: `strfunc.dissect2` subsection 14.13 on page 69

Called by: `checkterms.check_all` subsection 11.5 on page 52

11.8. Function if_dequalify As Boolean

whether one of the dequalifying terms is present in the text

Arguments: `pos` – Long
`checkstring` – String

Subs and functions called: `strfunc.dissect2` subsection 14.13 on page 69

Called by: `checkterms.check_all` subsection 11.5 on page 52

12. Module list

12.1. User-defined data types

`termlist`

Data elements:
`Termref(maxtermlist)` – Long
`words(maxtermlist)` – String
`score(maxtermlist)` – Single (*added 15 Jan 2004*)
`num` – Long (*number of terms in termlist*)

12.2. Global variables and constants

Const **`maxtermlist`** = 50 (*maximum number of terms to consider*)
Const **`threshold_high`** = 91 (*(for readscore - don't analyse further)*)
Const **`threshold`** = 87 (*(for readscore - minimum)*)

12.3. Function bestmatch As String

modified 31 Jan 06. Output is the termref of the best Read term match and associated readscore

Arguments: **`pd_start`** – Long
`pd_fin` – Long
`debug_` – Boolean (Optional)

Subs and functions called: **`terms.exact_read_termref`** subsection 16.6 on page 75
`pd.part_nopunc` subsection 13.16 on page 61
`pd.text` subsection 13.25 on page 64
`list.getlist` subsection 12.8 on page 56
`list.display` subsection 12.6 on page 55
`list.expand` subsection 12.4 on page 54

Called by: **`freetext_core.analyse_pd`** subsection 9.8 on page 48

12.4. Function expand As termlist

For each term in in_list, generate a new term by using abbreviations or synonyms Search maximum 5 words at a time, starting with longer matches can expand from s1–>s2 but not the other way round because s1 words might be more specific

Arguments: **`in_list`** – termlist
`pd_start` – Long (Optional)
`pd_fin` – Long (Optional)
`leeway` – Long (Optional)

Subs and functions called: **checkterms.in_list** subsection 11.6 on page 53
strfunc.numwords subsection 14.7 on page 67
strfunc.words subsection 14.4 on page 66
synonym.s1_pos subsection 15.11 on page 73
synonym.s1_priority subsection 15.14 on page 74
synonym.s2 subsection 15.13 on page 74
list.getlist subsection 12.8 on page 56
list.add_termlists subsection 12.7 on page 55
synonym.s1 subsection 15.13 on page 74

Called by: **list.bestmatch** subsection 12.3 on page 54

12.5. Sub test

Arguments: **phrase** – String
pd_start – Long (Optional)
pd_fin – Long (Optional)
leeway – Long (Optional)

Subs and functions called: **pd.max** subsection 13.28 on page 64
wordlist.init subsection 17.4 on page 78
terms.init subsection 16.4 on page 75
pd.init_read subsection 13.21 on page 63
list.display subsection 12.6 on page 55
list.getlist subsection 12.8 on page 56

Called by: **in_out.filepath** subsection 18.1 on page 83

12.6. Sub display

writes the contents of termlist to the debug window

Arguments: **in_list** – termlist

Subs and functions called: **checkterms.in_list** subsection 11.6 on page 53
in_out.read_term subsection 18.3 on page 83

Called by: **list.bestmatch** subsection 12.3 on page 54
list.test subsection 12.5 on page 55

12.7. Function add_termlists As termlist

Arguments: **t1** – termlist
t2 – termlist

Subs and functions called: none

Called by: `list.expand` subsection 12.4 on page 54

12.8. Function getlist As termlist

If no words retrieved, try with left/right removed (AFTERWARDS). Modified 20JUN05 so it does not use database tables. Leeway is either zero or 1 (if it is OK to select terms which have one more word than 'words') – now only using leeway=0 (sub bestmatch modified) Removal of ignorable words – this should be checked using remove_ignorable prior to using this function

Arguments: `words` – String (ByVal)
`pd_start` – Long (Optional)
`pd_fin` – Long (Optional)
`leeway` – Long (Optional)

Subs and functions called: `freetext_core.remove_ignorable` subsection 9.9 on page 49
`strfunc.numwords` subsection 14.7 on page 67
`wordlist.pos_singlewords` subsection 17.10 on page 79
`wordlist.pos_doublewords` subsection 17.11 on page 79
`freetext_core.readscore` subsection 9.10 on page 49
`wordlist.wordlist_termref` subsection 17.15 on page 80
`strfunc.words` subsection 14.4 on page 66
`wordlist.dbl_termref` subsection 17.13 on page 80
`wordlist.termref_in_doublewords1` subsection 17.18 on page 81
`wordlist.termref_in_doublewords2` subsection 17.18 on page 81

Called by: `list.bestmatch` subsection 12.3 on page 54
`list.expand` subsection 12.4 on page 54
`list.test` subsection 12.5 on page 55

12.9. Function wordtermrefs_count As Long

Arguments: `word` – String

Subs and functions called: `wordlist.pos_singlewords` subsection 17.10 on page 79

Called by: none

13. Module pd

Partdata module. Similar to the old partdata module (used for the dosage analyser) but it stores punctuation separately and there is an extra section for attributes

13.1. Global variables and constants

Const **maxpartdata** = 1000
partdata_used – Long
partdata(maxpartdata) – String
punc(maxpartdata) – String (*punctuation*)
attrib(maxpartdata) – String (*attribute ' e.g. negative, family etc.*)
meaning(maxpartdata) – String (*meaning*)

13.2. Sub check_compressed

Use AFTER sub compress. If date_only, removes all results except the first full date - NO LONGER, now if >1 date –> machinequery). Converts duration weeks and gestation into 'LABS' - gest Checks that only one gestational age. Checks that sysbp greater than diabp. Checks that dateprev, datenext refer to a clinical event

Arguments: **maybe_pregnant** – Boolean (Optional)
labtest – String (Optional)

Subs and functions called: **strfunc.words** subsection 14.4 on page 66
pd.Attr subsection 13.9 on page 59
pd.remove subsection 13.24 on page 63
pd.set_attr subsection 13.12 on page 60
terms.true_term subsection 16.5 on page 75
strfunc.dissect2 subsection 14.13 on page 69
pd.set_mean subsection 13.13 on page 60
strfunc.in_set subsection 14.5 on page 66
pd.mean subsection 13.10 on page 59
pd.remove_from_compressed subsection 13.3 on page 57

Called by: **freetext_core.main** subsection 9.5 on page 47

13.3. Sub remove_from_compressed

removes all entries with a certain attribute (lmp etc.) if there is a risk it might be wrong.

Arguments: **attr_to_remove** – String (Optional) (ByVal)
type_to_remove – String (Optional) (ByVal)

Subs and functions called: **pd.remove** subsection 13.24 on page 63
strfunc.dissect2 subsection 14.13 on page 69
pd.mean subsection 13.10 on page 59

Called by: **pd.check_compressed** subsection 13.2 on page 57

13.4. Sub compress

Converts pd into a single list of entries. Used at the end of interpretation.

Arguments: none

Subs and functions called: `pd.Attr` subsection 13.9 on page 59
`strfunc.in_set` subsection 14.5 on page 66
`pd.mean` subsection 13.10 on page 59
`pd.set_mean` subsection 13.13 on page 60
`pd.correct_attr` subsection 13.5 on page 58
`pd.set_attr` subsection 13.12 on page 60
`pd.remove` subsection 13.24 on page 63

Called by: `freetext_core.main` subsection 9.5 on page 47

13.5. Function correct_attr As Boolean

whether the attribute at this position is appropriate

Arguments: `pos` – Long

Subs and functions called: `strfunc.dissect2` subsection 14.13 on page 69
`pd.mean` subsection 13.10 on page 59
`strfunc.in_set` subsection 14.5 on page 66
`pd.Attr` subsection 13.9 on page 59

Called by: `pd.compress` subsection 13.4 on page 58

13.6. Sub show_all

prints the whole of pd to the debug window - (not used 23jun05)

Arguments: none

Subs and functions called: none

Called by: none

13.7. Sub show_all_2

prints the whole of pd to the debug window

Arguments: none

Subs and functions called: none

Called by: `freetext_core.main` subsection 9.5 on page 47

13.8. Function true_ As Long)

Arguments: `pos` – Long

Subs and functions called: `pd.Attr` subsection 13.9 on page 59

Called by: `freetext_core.readscore` subsection 9.10 on page 49

13.9. Function Attr As String

Arguments: `pos` – Long

Subs and functions called: none

Called by: `freetext_core.main_termref` subsection 9.4 on page 46
`freetext_core.attrib_search` subsection 9.7 on page 48
`freetext_core.analyse_pd` subsection 9.8 on page 48
`freetext_core.readscore` subsection 9.10 on page 49
`checkterms.check_all` subsection 11.5 on page 52
`pd.check_compressed` subsection 13.2 on page 57
`pd.compress` subsection 13.4 on page 58
`pd.correct_attr` subsection 13.5 on page 58
`pd.true_` subsection 13.8 on page 59
`synonym.trylink_2` subsection 15.9 on page 73
`terms.init` subsection 16.4 on page 75
`in_out.results_output` subsection 18.6 on page 84
`in_out.do_text_file` subsection 18.7 on page 85
`maintenance.delete_long_terms` subsection 19.7 on page 88
`maintenance.make_wordlist` subsection 19.10 on page 88
`maintenance.process_termlist` subsection 19.15 on page 90

13.10. Function mean As String

Arguments: `pos` – Long

Subs and functions called: none

Called by: `freetext_core.main_termref` subsection 9.4 on page 46
`freetext_core.attrib_search` subsection 9.7 on page 48
`freetext_core.analyse_pd` subsection 9.8 on page 48
`checkterms.check_all` subsection 11.5 on page 52
`pd.check_compressed` subsection 13.2 on page 57
`pd.remove_from_compressed` subsection 13.3 on page 57
`pd.compress` subsection 13.4 on page 58
`pd.correct_attr` subsection 13.5 on page 58

in_out.results_output subsection 18.6 on page 84
in_out.do_text_file subsection 18.7 on page 85

13.11. Sub del_attr

Arguments: **pos** – Long

Subs and functions called: none

Called by: none

13.12. Sub set_attr

Debug.Print "Set attribute: " partdata(pos) punc(pos) " " mean(pos) " " attr(pos) " -> " new_attribute If new_attribute <> "" Then Stop

Arguments: **new_attribute** – String
pos – Long

Subs and functions called: none

Called by: **freetext_core.attrib_search** subsection 9.7 on page 48
freetext_core.analyse_pd subsection 9.8 on page 48
attrib.pd_search2 subsection 10.6 on page 51
checkterms.check_all subsection 11.5 on page 52
pd.check_compressed subsection 13.2 on page 57
pd.compress subsection 13.4 on page 58

13.13. Sub set_mean

Arguments: **new_meaning** – String
pos – Long

Subs and functions called: none

Called by: **freetext_core.analyse_pd** subsection 9.8 on page 48
attrib.pd_search2 subsection 10.6 on page 51
pd.check_compressed subsection 13.2 on page 57
pd.compress subsection 13.4 on page 58

13.14. Sub add_attr

if there is already an attribute of any type at this position, exit sub

Arguments: `new_attribute` – String
`pos_start` – Long
`pos_fin` – Long (Optional)
`ignore_if_already` – Boolean (Optional)

Subs and functions called: none

Called by: `freetext_core.initial_search` subsection 9.6 on page 47

13.15. Sub add_mean

if there is already an meaning of any type at this position, exit sub

Arguments: `new_meaning` – String
`pos_start` – Long
`pos_fin` – Long (Optional)
`ignore_if_already` – Boolean (Optional)

Subs and functions called: none

Called by: `freetext_core.initial_search` subsection 9.6 on page 47

13.16. Function part_nopunc As String

partdata text only (no punctuation)

Arguments: `start` – Long (Optional)
`fin` – Long (Optional) (ByVal)

Subs and functions called: `pd.max` subsection 13.28 on page 64

Called by: `freetext_core.initial_search` subsection 9.6 on page 47
`freetext_core.readscore` subsection 9.10 on page 49
`list.bestmatch` subsection 12.3 on page 54
`synonym.trylink_2` subsection 15.9 on page 73
`maintenance.make_std_term_with_attr` subsection 19.5 on page 87

13.17. Function part_punc_nospace As String

includes punctuation but no spaces either side of punctuation

Arguments: `start` – Long
`fin` – Long

Subs and functions called: `pd.max` subsection 13.28 on page 64

Called by: `freetext_core.initial_search` subsection 9.6 on page 47
`attrib.pd_search2` subsection 10.6 on page 51

13.18. Function matchpattern As Boolean

whether a set of up to 5 words or meanings (w1-w5) with punctuation (p1-p5) match a set of entries in partdata

Arguments: `partdata_pos` – Long
`w1` – String
`p1` – String
`w2` – String
`p2` – String
`w3` – String
`p3` – String
`w4` – String
`p4` – String
`w5` – String
`p5` – String

Subs and functions called: `pd.matchposition` subsection 13.19 on page 62

Called by: `attrib.pd_search2` subsection 10.6 on page 51

13.19. Function matchposition As Boolean

word can represent either partdata text, or meaning if enclosed in [] modification 25-Oct-05 to allow different data types and punctuation to be separated by |

Arguments: `partdata_pos` – Long
`word` – String (ByVal)
`punct` – String (ByVal)

Subs and functions called: `strfunc.dissect2` subsection 14.13 on page 69
`pd.matchoption` subsection 13.20 on page 62

Called by: `pd.matchpattern` subsection 13.18 on page 62

13.20. Function matchoption As Boolean

match meaning / words

Arguments: `partdata_pos` – Long
`word` – String (ByVal)
`punct` – String (ByVal)

Subs and functions called: `strfunc.dissect` subsection 14.13 on page 69
`strfunc.words` subsection 14.4 on page 66
`pd.text` subsection 13.25 on page 64

Called by: `pd.matchposition` subsection 13.19 on page 62

13.21. Sub init_read

initialises partdata and punc using instring. also converts: + –> 'and', –> 'fracture'

Arguments: `instring` – String

Subs and functions called: `pd.clear` subsection 13.23 on page 63
`pd.st_type` subsection 13.22 on page 63
`strfunc.is_numeric` subsection 14.14 on page 70

Called by: `freetext_core.main` subsection 9.5 on page 47
`list.test` subsection 12.5 on page 55
`maintenance.read_attribute` subsection 19.3 on page 86
`maintenance.make_std_term_with_attr` subsection 19.5 on page 87

13.22. Function st_type As Long

Arguments: `instring` – String

Subs and functions called: `strfunc.is_text` subsection 14.6 on page 67
`strfunc.is_numeric` subsection 14.14 on page 70

Called by: `pd.init_read` subsection 13.21 on page 63

13.23. Sub clear

Arguments: none

Subs and functions called: none

Called by: `pd.init_read` subsection 13.21 on page 63

13.24. Sub remove

removes data from specified positions

Arguments: `pos1` – Long
`pos2` – Long (Optional)

Subs and functions called: none

Called by: `freetext_core.main_termref` subsection 9.4 on page 46
`checkterms.check_all` subsection 11.5 on page 52
`pd.check_compressed` subsection 13.2 on page 57
`pd.remove_from_compressed` subsection 13.3 on page 57
`pd.compress` subsection 13.4 on page 58

13.25. Function text As String

Arguments: `position` – Long

Subs and functions called: none

Called by: `freetext_core.initial_search` subsection 9.6 on page 47
`freetext_core.attrib_search` subsection 9.7 on page 48
`freetext_core.analyse_pd` subsection 9.8 on page 48
`freetext_core.readscore` subsection 9.10 on page 49
`attrib.pd_search2` subsection 10.6 on page 51
`list.bestmatch` subsection 12.3 on page 54
`pd.matchoption` subsection 13.20 on page 62
`wordlist.init_ignore` subsection 17.5 on page 78
`in_out.input_string` subsection 18.2 on page 83
`in_out.import_input_table` subsection 18.4 on page 84
`in_out.do_input_table` subsection 18.5 on page 84

13.26. Sub set_text

Arguments: `new_text` – String
`position` – Long

Subs and functions called: none

Called by: `freetext_core.initial_search` subsection 9.6 on page 47

13.27. Function punct As String

Arguments: `position` – Long

Subs and functions called: none

Called by: `freetext_core.attrib_search` subsection 9.7 on page 48
`freetext_core.analyse_pd` subsection 9.8 on page 48

13.28. Function max As Long

Arguments: none

Subs and functions called: none

Called by: `freetext_core.initial_search` subsection 9.6 on page 47
`freetext_core.attrib_search` subsection 9.7 on page 48
`freetext_core.analyse_pd` subsection 9.8 on page 48
`attrib.pd_search2` subsection 10.6 on page 51

checkterms.check_all subsection 11.5 on page 52
list.test subsection 12.5 on page 55
pd.part_nopunc subsection 13.16 on page 61
pd.part_punc_nospace subsection 13.17 on page 61
pd.part_punc subsection 13.29 on page 65
in_out.results_output subsection 18.6 on page 84
in_out.do_text_file subsection 18.7 on page 85

13.29. Function part_punc As String

includes punctuation with a space either side of punctuation

Arguments: **start** – Long
fin – Long

Subs and functions called: **pd.max** subsection 13.28 on page 64

Called by: **maintenance.read_attribute** subsection 19.3 on page 86

14. Module strfunc

Various functions for manipulating strings

14.1. Global variables and constants

Const **max_wd** = 30

14.2. Function get_date As String

attempts to convert a string into a date

Arguments: **s** – String
get_time – Boolean (Optional)

Subs and functions called: **strfunc.in_set** subsection 14.5 on page 66
strfunc.dissect2 subsection 14.13 on page 69
strfunc.get_date_average subsection 14.3 on page 66

Called by: **freetext_core.initial_search** subsection 9.6 on page 47

14.3. Function get_date_average As String

provides a replacement for first number (s1) from phrases such as 2-3 weeks, 5-6 days etc. average duration is used, rounded UP (no decimal places).

Arguments: `s1` – String
`s2` – String

Subs and functions called: none

Called by: `strfunc.get_date` subsection 14.2 on page 65

14.4. Function words As String

tested 31 dec 03 assume one space between words, and no spaces at the beginning

Arguments: `phrase` – String (ByVal)
`start` – Long
`numwd` – Long (Optional)
`finish` – Long (Optional)

Subs and functions called: `strfunc.dissect2` subsection 14.13 on page 69
`strfunc.numwords` subsection 14.7 on page 67

Called by: `freetext_core.analyse_pd` subsection 9.8 on page 48
`freetext_core.readscore` subsection 9.10 on page 49
`list.expand` subsection 12.4 on page 54
`list.getlist` subsection 12.8 on page 56
`pd.check_compressed` subsection 13.2 on page 57
`pd.matchoption` subsection 13.20 on page 62
`synonym.trylink_2` subsection 15.9 on page 73
`wordlist.init` subsection 17.4 on page 78
`wordlist.pos_doublewords` subsection 17.11 on page 79
`in_out.results_output` subsection 18.6 on page 84
`in_out.do_text_file` subsection 18.7 on page 85
`maintenance.read_attribute` subsection 19.3 on page 86
`maintenance.make_wordlist` subsection 19.10 on page 88

14.5. Function in_set As Boolean

whether target is one of a, b, c, d, e etc. stops when it encounters the first empty string

Arguments: `Target` – String
`a` – String
`b` –
`c` – String (Optional)
`d` – String (Optional)

e – String (Optional)
f – String (Optional)
g – String (Optional)
h – String (Optional)
i – String (Optional)
j – String (Optional)
k – String (Optional)
l – String (Optional)

Subs and functions called: none

Called by: `freetext_core.main_termref` subsection 9.4 on page 46
`freetext_core.attrib_search` subsection 9.7 on page 48
`freetext_core.analyse_pd` subsection 9.8 on page 48
`freetext_core.remove_ignorable` subsection 9.9 on page 49
`freetext_core.readscore` subsection 9.10 on page 49
`pd.check_compressed` subsection 13.2 on page 57
`pd.compress` subsection 13.4 on page 58
`pd.correct_attr` subsection 13.5 on page 58
`strfunc.get_date` subsection 14.2 on page 65
`strfunc.is_numeric` subsection 14.14 on page 70
`in_out.results_output` subsection 18.6 on page 84

14.6. Function is_text As Boolean

lower case only

Arguments: `instring` –

Subs and functions called: none

Called by: `pd.st_type` subsection 13.22 on page 63
`strfunc.phrase_match_pattern` subsection 14.15 on page 70
`maintenance.read_attribute` subsection 19.3 on page 86

14.7. Function numwords As Long

Arguments: `instring` – String (ByVal)

Subs and functions called: none

Called by: `freetext_core.remove_ignorable` subsection 9.9 on page 49
`freetext_core.readscore` subsection 9.10 on page 49
`list.expand` subsection 12.4 on page 54
`list.getlist` subsection 12.8 on page 56
`strfunc.words` subsection 14.4 on page 66
`strfunc.is_acronym` subsection 14.8 on page 68

strfunc.phrase_match_pattern subsection 14.15 on page 70
synonym.add subsection 15.6 on page 72
synonym.add_with_acronym subsection 15.7 on page 72
synonym.trylink_2 subsection 15.9 on page 73
maintenance.read_attribute subsection 19.3 on page 86

14.8. Function is_acronym As Boolean

whether or not abbrev is an acronym of full abbrev can either have spaces or no spaces

Arguments: **abbrev** – String (ByVal)
full – String

Subs and functions called: **strfunc.numwords** subsection 14.7 on page 67
strfunc.dissect subsection 14.13 on page 69

Called by: **synonym.add_with_acronym** subsection 15.7 on page 72

14.9. Function all_punc As Boolean

whether a string is entirely punctuation

Arguments: **s** – String

Subs and functions called: none

Called by: none

14.10. Function matchindex As Single

gives the percentage of larger word which smaller word matches

Arguments: **word1** – String
word2 – String

Subs and functions called: **strfunc.num_diff_char** subsection 14.11 on page 68

Called by: none

14.11. Function num_diff_char As Long

tested 3 feb 04 counts the number of characters which are different between str1 and str2 only considers up to the length of the shorter string, and only up to 3 different

Arguments: **str1** – String
str2 – String

Subs and functions called: none

Called by: `strfunc.matchindex` subsection 14.10 on page 68

14.12. Function dissect As String

rewritten 1-Oct-05 to use the VBA.split function via 'dissect2'. These functions are identical apart from the order of the arguments.

Arguments: `in_string` – String
`number` – Long
`delimiter` – String (Optional)

Subs and functions called: `strfunc.dissect2` subsection 14.13 on page 69

Called by: `pd.matchoption` subsection 13.20 on page 62
`strfunc.is_acronym` subsection 14.8 on page 68
`in_out.import_input_table` subsection 18.4 on page 84
`in_out.do_text_file` subsection 18.7 on page 85

14.13. Function dissect2 As String

if delimiter is not given it is assumed to be space

Arguments: `in_string` – String
`delimiter` – String (Optional)
`number` – Long (Optional)

Subs and functions called: none

Called by: `freetext_core.main_termref` subsection 9.4 on page 46
`freetext_core.remove_ignorable` subsection 9.9 on page 49
`freetext_core.readscore` subsection 9.10 on page 49
`attrib.pd_search2` subsection 10.6 on page 51
`checkterms.check_all` subsection 11.5 on page 52
`checkterms.if_qualify` subsection 11.7 on page 53
`checkterms.if_dequalify` subsection 11.8 on page 53
`pd.check_compressed` subsection 13.2 on page 57
`pd.remove_from_compressed` subsection 13.3 on page 57
`pd.correct_attr` subsection 13.5 on page 58
`pd.matchposition` subsection 13.19 on page 62
`strfunc.get_date` subsection 14.2 on page 65
`strfunc.words` subsection 14.4 on page 66
`strfunc.dissect` subsection 14.13 on page 69
`strfunc.phrase_match_pattern` subsection 14.15 on page 70
`synonym.sl_priority` subsection 15.14 on page 74

maintenance.read_attribute subsection 19.3 on page 86
maintenance.process_termlist subsection 19.15 on page 90

14.14. Function is_numeric As Boolean

determines whether a string contains only a single number or part of a single number if lab_results_mode is TRUE, words like 'normal', 'abnormal' etc. are considered to be numbers.

Arguments: **instring** – String
lab_results_mode – Boolean (Optional)

Subs and functions called: **strfunc.in_set** subsection 14.5 on page 66

Called by: **freetext_core.initial_search** subsection 9.6 on page 47
pd.init_read subsection 13.21 on page 63
pd.st_type subsection 13.22 on page 63

14.15. Function phrase_match_pattern As Long

*Whether or not strings 1 and 2 match (word by word) - used by maintenance.rm_attr =number, *=text, ?=anything - for a whole word*

Arguments: **instring** – String
pattern – String

Subs and functions called: **strfunc.numwords** subsection 14.7 on page 67
strfunc.dissect2 subsection 14.13 on page 69
strfunc.is_text subsection 14.6 on page 67

Called by: **maintenance.rm_attr** subsection 19.6 on page 87

15. Module synonym

Priority codes: 5 = exact match (both ways) e.g. chronic obstructive pulmonary disease = copd; 4 = almost exact match (both ways) e.g. cancer = malignant neoplasm; 3 = moderate match (s1 is) e.g. b pne = bronchopneumonia; e.g. carcinoma (is a type of) = malignant neoplasm; 2 = non-standard abbreviation or distorted form; possible one-way match (s2 wider than s1) e.g. rsi = repetitive strain injury; 1 = loosely associated (s2 wider than s1) e.g. foot (is a part of) = lower limb; -100 = opposite.

15.1. Global variables and constants

Const `maxsynonym` = 2000
`s_used` – Long
`s1_sorted(maxsynonym)` – String
`s1_result(maxsynonym)` – String (*priority and numwords, used for get_search_summary*)
`s1_s2(maxsynonym)` – String (*not sorted*)
`s2_sorted(maxsynonym)` – String (*first sort order*)
`s2_s2num(maxsynonym)` – Long (*2nd sort desc*)
`s2_s1num(maxsynonym)` – Long (*3rd sort desc*)
`s2_priority(maxsynonym)` – String (*4th sort desc*)
`s2_s1(maxsynonym)` – String (*not sorted*)

15.2. Sub infile

Inputs everything from filenumber 1

Arguments: none

Subs and functions called: none

Called by: `freetext_core.init_all` subsection 9.2 on page 45

15.3. Sub outfile

Outputs arrays to filenumber 1

Arguments: none

Subs and functions called: none

Called by: `freetext_core.outfile_all` subsection 9.3 on page 46

15.4. Sub init

initialises synonym arrays

Arguments: none

Subs and functions called: `synonym.s2` subsection 15.13 on page 74
`synonym.s1` subsection 15.13 on page 74

Called by: `freetext_core.outfile_all` subsection 9.3 on page 46

15.5. Sub del

deletes entry

Arguments: `s1` – String (ByVal)
`s2` – String (ByVal)

Subs and functions called: none

Called by: none

15.6. Sub add

adds a term to the synonym list updates priority if term already exists s1 and s2 are combined primary key - therefore there cannot be any duplicates

Arguments: `s1` – String (ByVal)
`s2` – String (ByVal)
`priority` – Long (Optional)
`bothways` – Boolean (Optional)

Subs and functions called: `strfunc.numwords` subsection 14.7 on page 67
`wordlist.init_ignore` subsection 17.5 on page 78
`freetext_core.remove_ignorable` subsection 9.9 on page 49

Called by: `synonym.add_with_acronym` subsection 15.7 on page 72

15.7. Sub add_with_acronym

automatically add spaced-out version of acronym (e.g. mi would also add m i)

Arguments: `s1` – String (ByVal)
`s2` – String (ByVal)
`priority` – Long (Optional)
`bothways` – Boolean (Optional)

Subs and functions called: `synonym.add` subsection 15.6 on page 72
`strfunc.is_acronym` subsection 14.8 on page 68
`strfunc.numwords` subsection 14.7 on page 67

Called by: none

15.8. Function get_search_summary As String

new version - requires arrays Call init ' for testing

Arguments: `instring` – String

Subs and functions called: none

Called by: `freetext_core.initial_search` subsection 9.6 on page 47

15.9. Function trylink_2 As String

NEW VERSION tries to match a Read term segment to pd (between pd_start and pd_fin) starts from the beginning of the Read term segment; tries to match the whole of pd between pd_start and pd_fin, then tries to get the largest possible match. If not possible, tries smaller segments of the Read term but always starting from the beginning. OUTPUT: priority position_within_pd_start position_within_pd_fin read_fin (space separated) MODIFIED 11 Dec 2005 to give an output with priority 6 if read_term_segment = pdstring

Arguments: `read_term_segment` – String (ByVal)
`pd_start` – Long
`pd_fin` – Long
`cur_true` – Boolean

Subs and functions called: `pd.part_nopunc` subsection 13.16 on page 61
`strfunc.numwords` subsection 14.7 on page 67
`strfunc.words` subsection 14.4 on page 66
`pd.Attr` subsection 13.9 on page 59
`synonym.s2_pos` subsection 15.11 on page 73

Called by: `freetext_core.readscore` subsection 9.10 on page 49

15.10. Function s2_pos As Long

Call init ' returns the first position of s2 text in s2 sorted list

Arguments: `s2_text` – String

Subs and functions called: none

Called by: `synonym.trylink_2` subsection 15.9 on page 73

15.11. Function s1_pos As Long

returns the first position of s1 text in s1 sorted list

Arguments: `s1_text` – String

Subs and functions called: none

Called by: `list.expand` subsection 12.4 on page 54

15.12. Function s2 As String

Arguments: `s1_pos` – Long

Subs and functions called: none

Called by: `list.expand` subsection 12.4 on page 54
`synonym.init` subsection 15.4 on page 71

15.13. Function s1 As String

Arguments: `s1_pos` – Long

Subs and functions called: none

Called by: `list.expand` subsection 12.4 on page 54
`synonym.init` subsection 15.4 on page 71

15.14. Function s1_priority As Long

Arguments: `s1_pos` – Long

Subs and functions called: `strfunc.dissect2` subsection 14.13 on page 69

Called by: `list.expand` subsection 12.4 on page 54

16. Module terms

Contains the list of Read terms for the purposes of the program

16.1. Global variables and constants

Const `max_usedterms` = 100000
Const `max_allterms` = 150000
`a_std_term(max_usedterms)` – String (*table of std_term (ordered) to get termref*)
`a_termref(max_usedterms)` – Long (*table of std_term (ordered) to get termref*)
`a_terms_used` – Long
`b_termref(max_allterms)` – Long (*to get std_term or attrib_str. All termrefs included*)
`b_std_term(max_allterms)` – String
`b_attrib_str(max_allterms)` – String
`b_type(max_allterms)` – String (*data type of Read term (pregnancy, labtest, death etc.)*)
`b_terms_used` – Long

16.2. Sub infile

Inputs everything from filenumber 1

Arguments: none

Subs and functions called: none

Called by: `freetext_core.init_all` subsection 9.2 on page 45

16.3. Sub outfile

Outputs arrays to filenumber 1

Arguments: none

Subs and functions called: none

Called by: `freetext_core.outfile_all` subsection 9.3 on page 46

16.4. Sub init

Arguments: none

Subs and functions called: `terms.std_term` subsection 16.8 on page 76
`pd.Attr` subsection 13.9 on page 59

Called by: `freetext_core.outfile_all` subsection 9.3 on page 46
`list.test` subsection 12.5 on page 55
`maintenance.delete_superfluous_terms` subsection 19.8 on page 88

16.5. Function true_term As Boolean

whether a term contains a true part

Arguments: `Termref` – Long

Subs and functions called: `terms.attrib_str` subsection 16.9 on page 76

Called by: `pd.check_compressed` subsection 13.2 on page 57

16.6. Function exact_read_termref As Long

attempts to find an exact match to Read (using std_terms) – returns termref NB terms in std_term column have spaces before and after the words

Arguments: `search_term` – String

Subs and functions called: none

Called by: `list.bestmatch` subsection 12.3 on page 54
`maintenance.find_similar_term` subsection 19.9 on page 88

16.7. Function read_type As String

returns the type code of the Read Term (whether pregnancy, death, labtest etc.)

Arguments: `Termref` – Long

Subs and functions called: none

Called by: `freetext_core.main_termref` subsection 9.4 on page 46

16.8. Function std_term As String

standardised term for a termref

Arguments: `Termref` – Long

Subs and functions called: none

Called by: `freetext_core.main_termref` subsection 9.4 on page 46
`freetext_core.readscore` subsection 9.10 on page 49
`terms.init` subsection 16.4 on page 75
`maintenance.delete_superfluous_terms` subsection 19.8 on page 88
`maintenance.make_wordlist` subsection 19.10 on page 88
`maintenance.process_termlist` subsection 19.15 on page 90
`maintenance.expand_rightleft` subsection 19.16 on page 90

16.9. Function attrib_str As String

attribute string for a termref

Arguments: `Termref` – Long

Subs and functions called: none

Called by: `freetext_core.readscore` subsection 9.10 on page 49
`terms.true_term` subsection 16.5 on page 75

17. Module wordlist

3 tables for rapid lookup

17.1. Global variables and constants

Const **maxsingle** = 200000
Const **maxdouble** = 300000
Const **maxwords** = 100000
Const **maxignore** = 100
s_termref(maxsingle) – Long
s_words(maxsingle) – String
s_numwd(maxsingle) – Byte
s_max – Long
d_termref(maxdouble) – Long
d_words(maxdouble) – String
d_numwd(maxdouble) – Byte
d_max – Long
w_words(maxwords) – String
w_clinical(maxwords) – Boolean (*added 30jan06; whether the word is possibly part of a Clinical Term*)
w_top(40) – Long (*start position for words of different lengths*)
w_max – Long
ignorelist(maxignore) – String (*words which can be ignored e.g. if, and, of, the*)
ignorelistnum – Long
ignorephrase(maxignore) – String (*words which can be ignored e.g. if, and, of, the*)
ignorephrasenum – Long

17.2. Sub infile

Inputs everything from filenumber 1

Arguments: none

Subs and functions called: none

Called by: **freetext_core.init_all** subsection 9.2 on page 45

17.3. Sub outfile

Outputs arrays to filenumber 1

Arguments: none

Subs and functions called: none

Called by: **freetext_core.outfile_all** subsection 9.3 on page 46

17.4. Sub init

initialises the wordlist arrays

Arguments: none

Subs and functions called: `strfunc.words` subsection 14.4 on page 66
`wordlist.init_ignore` subsection 17.5 on page 78

Called by: `freetext_core.outfile_all` subsection 9.3 on page 46
`list.test` subsection 12.5 on page 55

17.5. Sub init_ignore

IGNORABLE LIST

Arguments: none

Subs and functions called: `pd.text` subsection 13.25 on page 64

Called by: `synonym.add` subsection 15.6 on page 72
`wordlist.init` subsection 17.4 on page 78
`maintenance.init_read_attr_tables` subsection 19.2 on page 86
`maintenance.process_termlist` subsection 19.15 on page 90

17.6. Function in_wordlist As String

returns CLIN or WORD depending whether the word is clinical whether a word is in the wordlist list (sorted by wordlength, then word)

Arguments: `instring` – String

Subs and functions called: none

Called by: `wordlist.in_wordlist_OLD` subsection 17.7 on page 78
`wordlist.wordsearch` subsection 17.19 on page 81

17.7. Function in_wordlist_OLD As Boolean

whether a word is in the wordlist list (sorted by wordlength, then word)

Arguments: `instring` – String

Subs and functions called: `wordlist.in_wordlist` subsection 17.7 on page 78

Called by: none

17.8. Function approx_wordlist As Long

approximate position of a word in the wordlist list (sorted by wordlength, then word)

Arguments: `instring` – String

Subs and functions called: none

Called by: `wordlist.wordsearch` subsection 17.19 on page 81

17.9. Function pos_wordlist As Long

chooses either singlewords or doublewords depending on number of words in instring instring must contain either one or two words.

Arguments: `search_top` – Boolean
`instring` – String
`min_numwd` – Long
`max_numwd` – Long

Subs and functions called: `wordlist.pos_doublewords` subsection 17.11 on page 79
`wordlist.pos_singlewords` subsection 17.10 on page 79

Called by: none

17.10. Function pos_singlewords As Long

position of first or last termref in singlewords. If result zero, the word is not in singlewords search_top = True means look for the top one

Arguments: `search_top` – Boolean
`instring` – String
`min_numwd` – Long
`max_numwd` – Long

Subs and functions called: none

Called by: `list.getlist` subsection 12.8 on page 56
`list.wordtermrefs_count` subsection 12.9 on page 56
`wordlist.pos_wordlist` subsection 17.9 on page 79
`wordlist.termref_in_singlewords` subsection 17.16 on page 81

17.11. Function pos_doublewords As Long

position of first or last termref in doublewords. If result zero, the word is not in doublewords search_top = True means look for the top one

Arguments: `search_top` – Boolean
`instring` – String (ByVal)
`min_numwd` – Long
`max_numwd` – Long

Subs and functions called: `strfunc.words` subsection 14.4 on page 66

Called by: `list.getlist` subsection 12.8 on page 56
`wordlist.pos_wordlist` subsection 17.9 on page 79
`wordlist.termref_in_doublewords1` subsection 17.18 on page 81
`wordlist.termref_in_doublewords2` subsection 17.18 on page 81

17.12. Function sng_termref As Long

Arguments: `pos` – Long

Subs and functions called: none

Called by: none

17.13. Function dbl_termref As Long

Arguments: `pos` – Long

Subs and functions called: none

Called by: `list.getlist` subsection 12.8 on page 56

17.14. Function dbl_numwd As Long

Arguments: `pos` – Long

Subs and functions called: none

Called by: none

17.15. Function wordlist_termref As Long

if numwd greater then 1, uses doublewords dictionary

Arguments: `pos` – Long
`numwd` – Long

Subs and functions called: none

Called by: `list.getlist` subsection 12.8 on page 56

17.16. Function termref_in_singlewords As Boolean

Whether a termref appears in a defined list within singlewords top and bot must be at the top or bottom of a list with numwd

Arguments: `Termref` – Long
`top` – Long
`bot` – Long

Subs and functions called: `wordlist.pos_singlewords` subsection 17.10 on page 79

Called by: none

17.17. Function termref_in_doublewords1 As Boolean

Whether a termref appears in a defined list within doublewords There are two copies of this function with different static variables, enabling fast searching with two different parts of the list. top and bot must be at the top or bottom of a list with numwd

Arguments: `Termref` – Long
`top` – Long
`bot` – Long

Subs and functions called: `wordlist.pos_doublewords` subsection 17.11 on page 79

Called by: `list.getlist` subsection 12.8 on page 56

17.18. Function termref_in_doublewords2 As Boolean

Whether a termref appears in a defined list within doublewords top and bot must be at the top or bottom of a list with numwd

Arguments: `Termref` – Long
`top` – Long
`bot` – Long

Subs and functions called: `wordlist.pos_doublewords` subsection 17.11 on page 79

Called by: `list.getlist` subsection 12.8 on page 56

17.19. Function wordsearch As String

tries to convert a word into a standard form (or without spelling mistakes) which is in wordlist returns CLIN (for a clinical word) or WORD (for any other word) followed by the correctly spelled word blank if the spelling cannot be corrected

Arguments: **word** – String (ByVal)
do_spellcheck – Boolean (Optional)

Subs and functions called: **wordlist.in_wordlist** subsection 17.7 on page 78
wordlist.approx_wordlist subsection 17.8 on page 79
freetext_core.fuzzylink subsection 9.11 on page 50

Called by: **freetext_core.initial_search** subsection 9.6 on page 47

17.20. Function ignorable As Boolean

whether or not a word is in the ignorable list for Read matching

Arguments: **instring** – String

Subs and functions called: none

Called by: **freetext_core.initial_search** subsection 9.6 on page 47
freetext_core.remove_ignorable subsection 9.9 on page 49
freetext_core.readscore subsection 9.10 on page 49
maintenance.read_attribute subsection 19.3 on page 86

17.21. Function ignore_max As ignore_max()

Arguments: none

Subs and functions called: none

Called by: **maintenance.read_attribute** subsection 19.3 on page 86

17.22. Function ignore_words As String

Arguments: **pos** – Long

Subs and functions called: none

Called by: **maintenance.read_attribute** subsection 19.3 on page 86

17.23. Function remove_ignore_phrases As String

removes phrases which are found in 'ignorable' list

Arguments: **instring** – String

Subs and functions called: **wordlist.initial_process** subsection 17.24 on page 83

Called by: `freetext_core.main` subsection 9.5 on page 47
`maintenance.read_attribute` subsection 19.3 on page 86
`maintenance.make_std_term_with_attr` subsection 19.5 on page 87

17.24. Function initial_process As String

pasting result of cervical smear

Arguments: `instring` – String

Subs and functions called: none

Called by: `wordlist.remove_ignore_phrases` subsection 17.23 on page 82

18. Module in_out

18.1. Function filepath As filepath()

Modified test program 02/06/04 - analyses a single text. Uses input and output tables

Arguments: none

Subs and functions called: `list.test` subsection 12.5 on page 55
`in_out.input_string` subsection 18.2 on page 83
`freetext_core.init_all` subsection 9.2 on page 45
`freetext_core.main` subsection 9.5 on page 47
`freetext_core.main_termref` subsection 9.4 on page 46
`in_out.results_output` subsection 18.6 on page 84

Called by: none

18.2. Function input_string As String

Arguments: `id` – Long

Subs and functions called: `pd.text` subsection 13.25 on page 64

Called by: `in_out.filepath` subsection 18.1 on page 83

18.3. Function read_term As String

returns the actual Read or OXMIS term (not std_term) for a termref

Arguments: `Termref` – Long

Subs and functions called: none

Called by: **list.display** subsection 12.6 on page 55
in_out.results_output subsection 18.6 on page 84

18.4. Sub import_input_table

imports texts from a tab delimited table id, text (no text qualifier; no header row) deletes current input and output table

Arguments: **filepath** – String
text_col – Long
id_col – Long (Optional)
delimiter – String (Optional)
termref_col – Long (Optional)
has_header – Boolean (Optional)

Subs and functions called: **strfunc.dissect** subsection 14.13 on page 69
pd.text subsection 13.25 on page 64

Called by: none

18.5. Sub do_input_table

death, pregnancy are negative

Arguments: **with_termref** – Boolean (Optional)
death_ – Boolean (Optional)
pregnant_ – Boolean (Optional)
debug_ – Boolean (Optional)
append – Boolean (Optional)
labtest – String (Optional)
date_only – Boolean (Optional)
sicknote – Boolean (Optional)

Subs and functions called: **freetext_core.init_all** subsection 9.2 on page 45
freetext_core.main_termref subsection 9.4 on page 46
pd.text subsection 13.25 on page 64
freetext_core.main subsection 9.5 on page 47
in_out.results_output subsection 18.6 on page 84

Called by: none

18.6. Sub results_output

places the results in the output table

Arguments: `id` – Long
`debug_` – Boolean

Subs and functions called: `pd.max` subsection 13.28 on page 64
`strfunc.words` subsection 14.4 on page 66
`pd.mean` subsection 13.10 on page 59
`in_out.read_term` subsection 18.3 on page 83
`pd.Attr` subsection 13.9 on page 59
`strfunc.in_set` subsection 14.5 on page 66

Called by: `in_out.filepath` subsection 18.1 on page 83
`in_out.do_input_table` subsection 18.5 on page 84

18.7. Sub do_text_file

death, pregnancy are negative

Arguments: `infile` – String
`outfile` – String
`id_col` – Long
`text_col` – Long
`delimiter` – String (Optional)
`has_header` – Boolean (Optional)
`termref_col` – Long (Optional)
`death_` – Boolean (Optional)
`pregnant_` – Boolean (Optional)
`append` – Boolean (Optional)
`labtest` – String (Optional)
`date_only` – Boolean (Optional)
`sicknote` – Boolean (Optional)

Subs and functions called: `freetext_core.init_all` subsection 9.2 on page 45
`strfunc.dissect` subsection 14.13 on page 69
`freetext_core.main_termref` subsection 9.4 on page 46
`freetext_core.main` subsection 9.5 on page 47
`pd.max` subsection 13.28 on page 64
`strfunc.words` subsection 14.4 on page 66
`pd.mean` subsection 13.10 on page 59
`pd.Attr` subsection 13.9 on page 59

Called by: none

19. Module maintenance

19.1. Global variables and constants

`r1_raw_pattern(50)` – String (*read_attr1 table, stored as arrays for faster processing*)
`r1_position(50)` – String
`r1_replacement(50)` – String
`r1_num` – Long
`r2_pattern(100)` – String (*read_attr2 table, stored as arrays*)
`r2_attr(100)` – String
`r2_num` – Long

19.2. Sub init_read_attr_tables

imports all data from Read attribute tables into arrays

Arguments: none

Subs and functions called: **`wordlist.init_ignore`** subsection 17.5 on page 78

Called by: **`maintenance.read_attribute`** subsection 19.3 on page 86
`maintenance.make_std_term_with_attr` subsection 19.5 on page 87

19.3. Function read_attribute As String

MODIFIED 27 Oct 2005 - to read lookup patterns from database tables. input read_std_term. Generates a string characterising the important words of each Read term. e.g. TTIIFF means first two words are true, next two can be ignored, and last two are false. Results stored in the attr column of the terms table. Checking for ignorable and negative terms

Arguments: **`read_std_term`** – String (Optional)
`dont_init_pd` – Boolean (Optional)
`init_lookup` – Boolean (Optional)

Subs and functions called: **`maintenance.init_read_attr_tables`** subsection 19.2 on page 86
`pd.init_read` subsection 13.21 on page 63
`wordlist.remove_ignore_phrases` subsection 17.23 on page 82
`pd.part_punc` subsection 13.29 on page 65
`strfunc.numwords` subsection 14.7 on page 67
`strfunc.is_text` subsection 14.6 on page 67
`strfunc.dissect2` subsection 14.13 on page 69
`wordlist.ignorable` subsection 17.20 on page 82
`strfunc.words` subsection 14.4 on page 66
`wordlist.ignore_max` subsection 17.21 on page 82

`wordlist.ignore_words` subsection 17.22 on page 82
`maintenance.rm_attr` subsection 19.6 on page 87

Called by: `maintenance.make_std_term_with_attr` subsection 19.5 on page 87

19.4. Function make_std_term As String

New function for std_term - also converts a/n, c/o, h/o etc.

Arguments: `raw_term` – String (ByVal)
`init_lookup` – Boolean (Optional)

Subs and functions called: `maintenance.make_std_term_with_attr` subsection 19.5 on page 87

Called by: none

19.5. Function make_std_term_with_attr As String

New function for std_term - also converts a/n, c/o, h/o etc. converts a Read term to std_term format.

Arguments: `raw_term` – String (ByVal)
`init_lookup` – Boolean (Optional)

Subs and functions called: `maintenance.init_read_attr_tables` subsection 19.2 on page 86
`pd.init_read` subsection 13.21 on page 63
`wordlist.remove_ignore_phrases` subsection 17.23 on page 82
`maintenance.read_attribute` subsection 19.3 on page 86
`pd.part_nopunc` subsection 13.16 on page 61

Called by: `maintenance.make_std_term` subsection 19.4 on page 87
`maintenance.process_termlist` subsection 19.15 on page 90

19.6. Sub rm_attr

Output is the new attribute string. Instring is the Read term with punctuation

Arguments: `instring` – String
`pattern` – String
`cur_attr` – String
`new_attr` – String

Subs and functions called: `strfunc.phrase_match_pattern` subsection 14.15 on page 70

Called by: **maintenance.read_attribute** subsection 19.3 on page 86

19.7. Sub delete_long_terms

Deletes all terms with >6 non-ignorable words from 'terms' table and wordlist.

Arguments: none

Subs and functions called: **maintenance.term_remove_NEW** subsection 19.13 on page 89
pd.Attr subsection 13.9 on page 59

Called by: none

19.8. Sub delete_superfluous_terms

Deletes all superfluous terms from 'terms' table and wordlist.

Arguments: none

Subs and functions called: **terms.init** subsection 16.4 on page 75
terms.std_term subsection 16.8 on page 76
maintenance.term_remove_NEW subsection 19.13 on page 89
maintenance.find_similar_term subsection 19.9 on page 88

Called by: none

19.9. Function find_similar_term As Long

Returns the termref of a similar term without the or of an identical term

Arguments: **interm** – String
start_phrase – String (Optional)
end_phrase – String (Optional)

Subs and functions called: **terms.exact_read_termref** subsection 16.6 on page 75

Called by: **maintenance.delete_superfluous_terms** subsection 19.8 on page 88

19.10. Sub make_wordlist

Generates 3 tables: singlewords , doublewords, wordlist. Also generates the lookup table file.

Arguments: none

Subs and functions called: **terms.std_term** subsection 16.8 on page 76
pd.Attr subsection 13.9 on page 59
maintenance.count_t subsection 19.11 on page 89

`strfunc.words` subsection 14.4 on page 66
`freetext_core.outfile_all` subsection 9.3 on page 46

Called by: none

19.11. Function count_t As Integer

minimum number of words in attr_string

Arguments: `attr_string` – String

Subs and functions called: none

Called by: `maintenance.make_wordlist` subsection 19.10 on page 88

19.12. Sub term_remove_BATCH

comma separated list of termrefs

Arguments: `Termref_list` – String
`Comment` – String (Optional)

Subs and functions called: none

Called by: none

19.13. Sub term_remove_NEW

Arguments: `Termref` – Long
`Comment` – String (Optional)

Subs and functions called: none

Called by: `maintenance.delete_long_terms` subsection 19.7 on page 88
`maintenance.delete_superfluous_terms` subsection 19.8 on page 88

19.14. Function read_code_oxmis As String

Arguments: `oxmis_termref` – Long

Subs and functions called: none

Called by: `maintenance.process_termlist` subsection 19.15 on page 90

19.15. Sub process_termlist

Arguments: `blank_only` – Boolean (Optional)
`not_readcode` – Boolean (Optional)
`termlist` – String (Optional)

Subs and functions called: `wordlist.init_ignore` subsection 17.5 on page 78
`maintenance.read_code_oxmis` subsection 19.14 on page 89
`maintenance.make_std_term_with_attr` subsection 19.5 on page 87
`terms.std_term` subsection 16.8 on page 76
`pd.Attr` subsection 13.9 on page 59
`strfunc.dissect2` subsection 14.13 on page 69

Called by: none

19.16. Sub expand_rightleft

Processes termlist: converts l and lt to left, and vice versa for right; only if the termlist contains terms for BOTH RIGHT AND LEFT. Need to regenerate wordlists afterwards.

Arguments: none

Subs and functions called: `terms.std_term` subsection 16.8 on page 76

Called by: none
